# A Sequential Dual‐Model Strategy Based on Photoactivatable Metallopolymer for On‐Demand Release of Photosensitizers and Anticancer Drugs

**DOI:** 10.1002/advs.202103334

**Published:** 2021-10-18

**Authors:** Maomao He, Guangli He, Peiyuan Wang, Suhua Jiang, Ziyue Jiao, Dongmei Xi, Pengcheng Miao, Xuefei Leng, Zhiyong Wei, Yang Li, Yanjun Yang, Ran Wang, Jianjun Du, Jiangli Fan, Wen Sun, Xiaojun Peng

**Affiliations:** ^1^ State Key Laboratory of Fine Chemicals Liaoning key Laboratory of Polymer Science and Engineering School of Chemical Engineering Dalian University of Technology Dalian 116024 China; ^2^ CAS Key Laboratory of Design and Assembly of Functional Nanostructures Fujian Institute of Research on the Structure of Matter Chinese Academy of Sciences Fuzhou 350002 China; ^3^ Ningbo Institute of Dalian University of Technology Ningbo 315016 China

**Keywords:** chemo‐photodynamic therapy, metallopolymer, on‐demand drug release, ROS‐responsive, Ru complexes

## Abstract

The synergistic combination of chemotherapy and photodynamic therapy has attracted considerable attention for its enhanced antitumoral effects; however, it remains challenging to successfully delivery photosensitizers and anticancer drugs while minimizing drug leakage at off‐target sites. A red‐light‐activatable metallopolymer, Poly(Ru/PTX), is synthesized for combined chemo‐photodynamic therapy. The polymer has a biodegradable backbone that contains a photosensitizer Ru complex and the anticancer drug paclitaxel (PTX) via a singlet oxygen (^1^O_2_) cleavable linker. The polymer self‐assembles into nanoparticles, which can efficiently accumulate at the tumor sites during blood circulation. The distribution of the therapeutic agents is synchronized because the Ru complex and PTX are covalently conjugate to the polymer, and off‐target toxicity during circulation is also mostly avoided. Red light irradiation at the tumor directly cleaves the Ru complex and produces ^1^O_2_ for photodynamic therapy. Sequentially, the generated ^1^O_2_ triggers the breakage of the linker to release the PTX for chemotherapy. Therefore, this novel sequential dual‐model release strategy creates a synergistic chemo‐photodynamic therapy while minimizing drug leakage. This study offers a new platform to develop smart delivery systems for the on‐demand release of therapeutic agents in vivo.

## Graphical abstract

Amphiphilic metallopolymers, which have a biodegradable polycarbonate backbone that contains the photosensitizer Ru complex and the anticancer drug paclitaxel (PTX) via a singlet oxygen cleavable linker, self‐assemble into nanoparticles. The nanoparticles carry the therapeutic agents into tumor cells without drug leakage. Red light irradiation induces sequential release of the Ru complex and PTX for enhanced chemo‐photodynamic therapy.

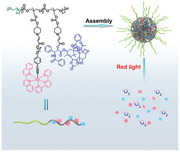

## 1 Introduction

Various therapies have been developed for the treatment of cancer, including chemotherapy, radiotherapy, chemodynamic therapy, and photodynamic therapy (PDT).^[^ ^1^ ^]^ However, treatment with a single therapy mode generally has limited efficacy due to the tumor heterogeneity.^[^ ^2^ ^]^ Thus, multimodal therapies that combine multiple treatments have been broadly exploited.^[^ ^1^, ^3^ ^]^ In particular, the combination of PDT with chemotherapy (chemo‐photodynamic therapy) presents unique advantages.^[^ ^4^ ^]^ Clinical data, which are supported by results from preclinical experiments using animals, have shown that PDT can be synergistically paired with chemotherapy to achieve promising therapeutic effects.^[^ ^5^ ^]^ PDT can effectively overcome drug resistance by sensitizing cancer cells for chemotherapy, activating the immune response, and preventing tumor angiogenesis. Additionally, PDT can help to reduce toxic side effects as well as the need for other anticancer drugs.^[^ ^6^ ^]^ Currently, PDT and chemotherapies are administered by simply combining photosensitizers with anticancer drugs. However, differences in the biodistribution and pharmacokinetics of the individual chemotherapeutic agents make it difficult to achieve efficient synergistic therapy.

Polymeric nanoparticles make promising nanocarriers in chemo‐photodynamic therapy due to their high biocompatibility and drug loading capacity.^[^ ^7^ ^]^ The biodistribution and pharmacokinetics of photosensitizers and anticancer drugs can be coordinated by co‐encapsulating the agents into the nanoparticles. Their release can be triggered by different stimuli including intracellular hypoxia, lysosome acidic environment, and overexpressed enzymes and reactive oxygen species (ROS).^[^ ^8^ ^]^ However, the drug release triggered by the intracellular stimuli would lead to uncontrollable drug release, because those stimuli are more or less present in normal cells.^[^ ^9^ ^]^ Moreover, photoresponsive nanocarries have also been designed for the combination PDT with chemotherapy.^[^ ^10^ ^]^ For instance, encapsulating both photosensitizers and anticancer drugs into ROS‐responsive polymeric nanoparticles could allow them to reach tumor sites simultaneously through enhanced penetration and retention (EPR) effects.^[^ ^8^, ^11^ ^]^ The ROS generated upon light irradiation not only results in PDT, but also causes the dissociation of the nanoparticle structures, triggering the release of both chemotherapeutic agents. Unfortunately, this formulation has an inevitable drug leakage during circulation and causes undesired side effects, which reduce the efficiency of synergistic therapy. Another commonly used strategy is to conjugate anticancer drugs to photosensitive semiconducting polymers with an ROS‐sensitive linkage.^[^ ^12^ ^]^ This design can prevent premature cargo leakage during blood circulation due to the covalent bond linker. However, the synthetic procedure of these polymers usually involves multistep reactions under the assistance of catalysts with a limited yield. Moreover, the non‐biodegradable polymer backbone makes them challenging for future clinical applications.^[^ ^12c^ ^]^ Therefore, it is urgent and necessary to develop new polymers with simple synthetic methods and excellent biodegradability for synergistic chemo‐photodynamic therapy.

**Figure 1:**
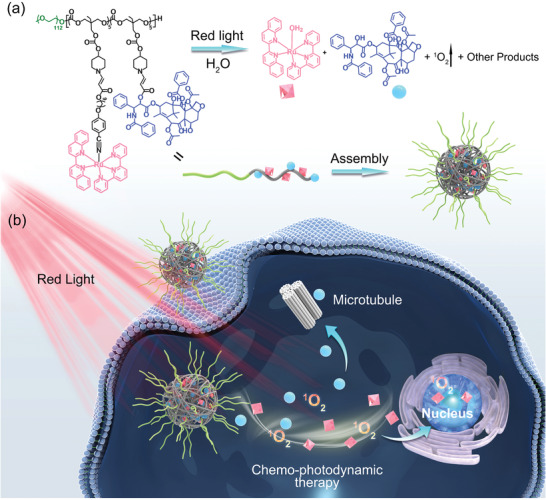
a) Chemical structure of the amphiphilic copolymer Poly(Ru/PTX). Red light irradiation induced cleavage of the Ru complex, generation of ^1^O_2_, and release of the anticancer drug PTX. b) Schematic illustration of self‐assembly, cell internalization, and chemo‐photodynamic therapy using Poly(Ru/PTX).

Herein, we report the synthesis of a novel photoactivatable metallopolymer, Poly(Ru/PTX), that can be remotely activated for combined chemo‐photodynamic therapy with red light irradiation (**Figure** **1**a). The polymer backbone was synthesized as a biodegradable and biocompatible block copolymer, consisting of methoxy polyethylene glycol (MPEG) and piperidine‐functionalized polycarbonate (PTMCP) moieties. Ru complexes and paclitaxel (PTX) were used as model photosensitizers and anticancer drugs, respectively. The introduction of photosensitizers and drugs at any desired ratio was easily realized via spontaneous amino‐alkynoate click reactions. The polymer self‐assembled into nanoparticles, which avoid undesired drug leakage while circulating in the bloodstream, because the photosensitizers and drugs are both covalently attached to the polymer networks. The polymer nanoparticles’ accumulation in the tumor site was due to the EPR effect. The red light irradiation of the tumor facilitates the direct release of Ru complexes and the production of singlet oxygen (^1^O_2_). The generated ^1^O_2_ then cleaves the ROS‐sensitive aminoacrylate linker leading to the release of PTX (Figure 1a).^[^ ^13^ ^]^ The photoactivable polymeric nanoparticle realizes the release of photosensitizers andaaanticancer drugs through the sequential dual‐model release strategy. Importantly, the novel polymer nanoparticles facilitate the combined chemo‐photodynamic therapy while minimizing drug leakage at off‐target sites in vivo (Figure 1b).

## 2 Results and Discussion

### 2.1 Preparation and Characterization

**Figure 2:**
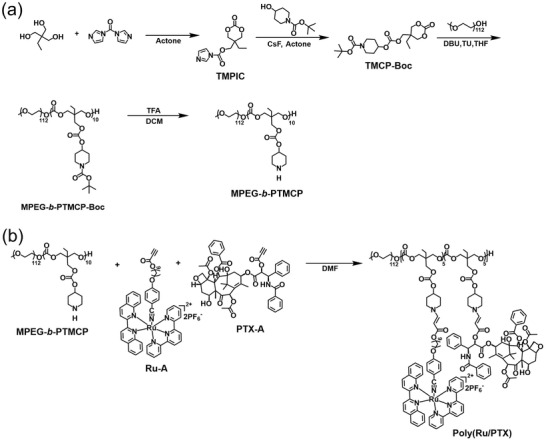
Synthetic route of Poly(Ru/PTX). a) The biodegradable polymer backbone MPEG‐*b*‐PTMCP was synthesized via the ring‐opening polymerization. b) Poly(Ru/PTX) was synthesized via spontaneous amino‐alkynoate click reaction.

**Figure 3:**
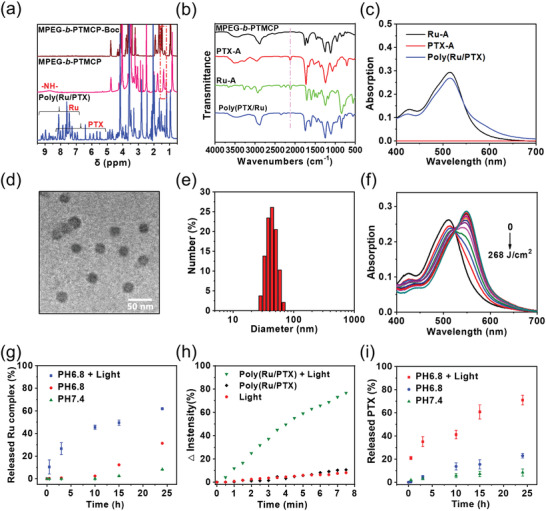
Preparation and physicochemical characterization of the Poly(Ru/PTX) nanoparticles. a) ^1^H NMR spectra of MPEG‐*b*‐PTMCP‐Boc, MPEG‐*b*‐PTMCP, and Poly(Ru/PTX). b) FT‐IR spectra of MPEG‐*b*‐PTMCP, PTX‐A, Ru‐A, and Poly(Ru/PTX). c) UV–vis spectra of PTX‐A, Ru‐A, and Poly(Ru/PTX). d) TEM image of Poly(Ru/PTX) nanoparticles. e) The diameter of Poly(Ru/PTX) nanoparticles measured using DLS. f) UV–vis spectra changes of the nanoparticles after 671 nm light irradiation for different doses. g) The percentages of released Ru complexes from Poly(Ru/PTX) nanoparticles under different conditions. h) The quantified intensity changes of DBPF at 453 nm in the fluorescence spectra. i) The percentages of released PTX from Poly(Ru/PTX) nanoparticles under different conditions.

Poly(Ru/PTX) was constructed through i) the synthesis of piperidine‐functionalized polyethylene glycol‐*block*‐polycarbonate (MPEG‐*b*‐PTMCP) (**Figure** 2a) and ii) the conjugation of Ru complexes and PTX to MPEG‐*b*‐PTMCP (Figure 2b). First, the piperidine‐appended cyclic carbonate monomer (TMCP‐Boc) was synthesized through the transesterification between trimethylol propane imidazole carbonate (TMPIC) and *tert*‐butyl 4‐hydroxypiperidine‐1‐carboxylate. The block copolymer MPEG‐*b*‐PTMCP‐Boc was synthesized via the organocatalyzed ring‐opening polymerization (ROP) of TMCP‐Boc with MPEG as the macroinitiator. After deprotection in the presence of trifluoroacetate (TFA), the proton peak corresponding to the *tert*‐butyl unit of the Boc group (1.45 ppm) completely disappeared, indicating that free secondary amine groups were activated (**Figure** 3a). The resulting proton peak of the secondary amine at *δ* 8.78 also indicated the removal of Boc groups.

According to the ^1^H nuclear magnetic resonance (NMR) integration results, the proton numbers of both polymers before and after deprotection were essentially unchanged except the disappearance of the Boc group, suggesting the stability of the polymer backbone (Figures S7 and S9, Supporting Information). MPEG‐*b*‐PTMCP further reacted with Ru complex alkynoate (Ru—A) and PTX alkynoate (PTX—A) through a spontaneous amine–alkynoate click reaction to yield Poly(Ru/PTX). For comparison, Poly(Ru) without PTX was also synthesized via conjugating Ru—A with the same polymer backbone MPEG‐*b*‐PTMCP (Figure S1, Supporting Information). All intermediates and the final polymer were then fully characterized using NMR, mass spectrometry (MS), and gel permeation chromatography (GPC) (Figures S3–S20, Supporting Information).

In the Fourier transform infrared (FT‐IR) spectra, the C≡C stretching vibration at 2110 cm^−1^ disappeared completely after a facile click reaction between alkynoates and secondary amines (Figure 3b). In addition, a characteristic metal‐to‐ligand charge transfer (MLCT) band of the Ru—A (400–700 nm) was exclusively present in the absorption spectrum of Poly(Ru/PTX) (Figure 3c), which further demonstrated the successful preparation of the polymer. Based on ^1^H NMR analysis, the molecular weight of Poly(Ru/PTX) was 18 kg mol^−1^, and each polymer chain contained five Ru complexes and five PTXs, which almost equaled their feeding ratios (Figure S19, Supporting Information). The excellent grafting yield could be attributed to the high reactivity of amino–alkynoate click reaction.^[^ ^14^ ^]^ Thus, this facile synthesis might be considered as an alternative and promising synthetic strategy for the preparation of polymer–drug conjugates, which should personalize cancer treatment by optimizing chemotherapy and the PDT.

In the prepared Poly(Ru/PTX), both the Ru complex and PTX are hydrophobic, while MPEG is highly hydrophilic. Thus, the polymer showed distinctly amphiphilic characteristics, which facilitated the self‐assembly of nanoparticles in aqueous solution with the MPEG serving as the outer shell, and the Ru complex and PTX‐functionalized polycarbonate serving as the hydrophobic inner core. The block copolymer assemblies were prepared by adding water to a tetrahydrofolate (THF) solution of Poly(Ru/PTX). Then, the organic solvent was removed via dialysis of the dispersion against water. The Poly(Ru/PTX) nanoparticles exhibited a monodispersed and uniform spherical morphology with a diameter of ≈26 nm in the transmission electron microscopy (TEM) image (Figure 3d). Dynamic light scattering (DLS) indicated that the hydrodynamic diameters of the nanoparticles were 34 nm, which was comparable to the TEM result (Figure 3e). Additionally, the Poly(Ru/PTX) nanoparticles were highly stable in aqueous solution, with no significant changes in the MLCT band or the average size observed in 48 h (Figures S23 and S24, Supporting Information). In contrast, free Ru—A was unstable in the aqueous solution after only 1 day of incubation because of the dissociation resulting from the spontaneous cleavage of the coordinated acetonitrile (Figure S27, Supporting Information).^[^ ^15^ ^]^ Importantly, the polymerization of Ru complexes improved the stability of the photosensitizer in the aqueous phase, which is beneficial for long blood circulation in vivo.

### 2.2 Photoresponsiveness and ROS‐Triggered Drug Release

Poly(Ru/PTX) nanoparticles showed a maximum MLCT band at 516 nm and extends to ≈750 nm (Figure S28, Supporting Information). Because red light can penetrate tissues much deeper than shorter wavelength, we used red light (671 nm, 200 mW cm^−2^) to activate Poly(Ru/PTX) nanoparticles. Red light irradiation redshifted the MLCT band from 516 to 554 nm (Figure 3f). Such a significant change in the absorption spectra was identical to that of the photocleavage of Ru—A (Figure S29, Supporting Information) and other similar Ru complexes.^[^ ^4^, ^16^ ^]^ The uncaged Ru[(tpy)(biq)(H_2_O)]^2+^ (Ru—H_2_O) exhibited its natural pink color after red light irradiation, further suggesting the release of Ru complexes (Figure S30, Supporting Information). The release percentage of Ru complexes was measured under physiological conditions (pH 7.4), and a tumor microenvironment (pH 6.8) (Figure 3g). In the pH 6.8 solution, up to 61.8% of the Ru complexes were released after light irradiation. Additionally, the release of Ru complexes decreased greatly without light irradiation. The cumulative drug release percentages were 31.4% at pH 6.8 and 8.2% at pH 7.4. Thus, the nanoparticles demonstrated excellent physiological stability and were able to release Ru complexes after irradiation. Next, the generation of ^1^O_2_ from Poly(Ru/PTX) under red light irradiation was confirmed by monitoring the fluorescence of the indicator 1,3‐diphenylisobenzofuran (DPBF). A gradual reduction in the fluorescence intensity of DPBF at 453 nm was observed for the Poly(Ru/PTX) solution, indicating the generation of ^1^O_2_ with red light irradiation (Figure 3h). After irradiating the above‐mentioned solutions for 7.5 min, the DPBF fluorescence intensity decreased by ≈80%. Similar results were also observed with Ru—H_2_O solution upon light irradiation (Figure S31, Supporting Information). Conversely, the fluorescence intensity of DPBF at 453 nm experienced no significant change, demonstrating that ^1^O_2_ was produced from the Ru complex containing nanoparticles. Although that the diffusion distance of ^1^O_2_ under physiological conditions is limited due to its short life time, it is expected that the released photosensitizers could significantly improve the PDT efficacy.^[^ ^17^ ^]^

In the presence of ^1^O_2_, the *β*‐aminoacrylate linkage undergoes fast oxidative degradation, which leads to the cleavage of ester linkage.^[^ ^13a^ ^]^ The efficient generation of ^1^O_2_ facilitated the fast release of PTX, which was confirmed using by high‐performance liquid chromatography (HPLC) analysis. After irradiating a solution of Poly(Ru/PTX) nanoparticles at red light irradiation (200 mW cm^−2^, 15 min), an elution peak identical to that of free PTX was observed at 9.6 min (Figure S32, Supporting Information). This peak, with a mass‐to‐charge ratio (*m*/*z*) of 853.9, also matched the molecular weight of PTX, suggesting that red light induced the release of free PTX (Figure S33, Supporting Information). Conversely, no elution peaks were observed for the Poly(Ru/PTX) nanoparticles lacking red light irradiation. We then studied in vitro red‐light‐triggered PTX release behavior (Figure 3i). It was found that whether experiencing an acidic tumor microenvironment (pH 6.8) or physiological conditions (pH 7.4), the release of PTX from Poly(Ru/PTX) nanoparticles was very low after 24 h of incubation without light irradiation. In contrast, the cumulative percentage of PTX released gradually increased to 71.1% when the irradiated Poly(Ru/PTX) nanoparticles were dialyzed.

We also performed GPC measurements to prove the photoinduced release of Ru complex and PTX (Figure S34, Supporting Information). Before light irradiation, the molecule weight of Poly(Ru/PTX) measured by GPC was 17 kg mol^−1^, which was comparable to that measured by ^1^H NMR. The delayed retention time of the Poly(Ru/PTX) after light irradiation indicated the cleavage of the Ru complexes and PTX from Poly(Ru/PTX). TEM observations further demonstrated that red light treatments contributed to nanoparticle morphological changes (Figure S35, Supporting Information). Together these findings demonstrate that Poly(Ru/PTX) nanoparticles could efficiently avoid drug leakage, thus reducing unintentional toxic side effects. Meanwhile, the phototriggered on‐demand drug delivery would ensure the tumor‐targeted therapeutic activity.

### 2.3 In Vitro Therapeutic Effect

**Figure 4:**
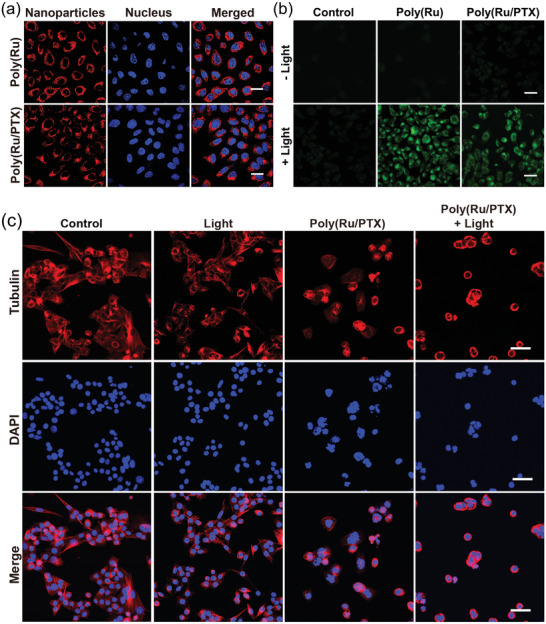
Intracellular distribution of phototriggered therapeutic agents released from Poly(Ru/PTX) nanoparticles. Confocal fluorescence images of a) MCF‐7 cells incubated with dye‐loaded nanoparticles for 6 h. Nuclei were stained with Hoechst 33342 (blue). b) MCF‐7 cells after 6 h treatments with the nanoparticles, followed by staining with H_2_DCFDA, with or without 671 nm laser irradiation (200 mW cm^−2^) for 15 min. Green fluorescence indicated the signals from 2',7'‐dichlorofluorescein (DCF). c) Microtubule staining of MCF‐7 cells after incubation with Poly(Ru/PTX) nanoparticles with or without light irradiation (671 nm, 200 mW cm^−2^, 15 min). Scale bar: 50 µm.

To evaluate the cellular uptake of nanoparticles, MCF‐7 cells were treated with dye‐loaded Poly(Ru) and Poly(Ru/PTX) nanoparticles for 6 h. The cells were then washed thoroughly and assessed using confocal laser scanning microscopy (CLSM). Obvious red fluorescence was observed from MCF‐7 cells incubated with dye‐loaded nanoparticles, which indicated the nanoparticles were effectively internalized by cancer cells (**Fi** **gure** **4a**. Since ^1^O_2_ was required to trigger the activation and release of PTX, intracellular ^1^O_2_ generation was directly studied using a cell‐permeable green fluorescent probe (2',7'‐dichlorodihydrofluorescein diacetate, H_2_DCFDA)as the indicator (Figure 4b). Green fluorescence signals were detected from cells that incubated with Poly(Ru) and Poly(Ru/PTX) nanoparticles after red light irradiation, while negligible fluorescence was detected in the other groups. These results clearly confirm that the red light irradiation of nanoparticles results in the intracellular generation of ^1^O_2_.

It is well known that PTX is a microtubule‐stabilizing agent that combats tumor growth by increasing microtubule polymerization and inducing mitotic arrest.^[^ ^18^ ^]^ We therefore observed the microtubule morphology of treated MCF‐7 cells via CLSM to evaluate the light‐controlled release of PTX inside cells (Figure 4c). The microtubule networks of MCF‐7 cells were well ordered and evenly distributed in the cytosol of control cells. The cells treated with Poly(Ru/PTX) nanoparticles showed some degree of microtubule aggregated morphology, while the microtubules of the cells treated with Poly(Ru/PTX) nanoparticles and red light irradiation displayed complete aggregation around the nucleus. Thus, the Poly(Ru/PTX) nanoparticles could be specifically activated by red light to generate ^1^O_2_ and further trigger the release of PTX.

**Figure 5:**
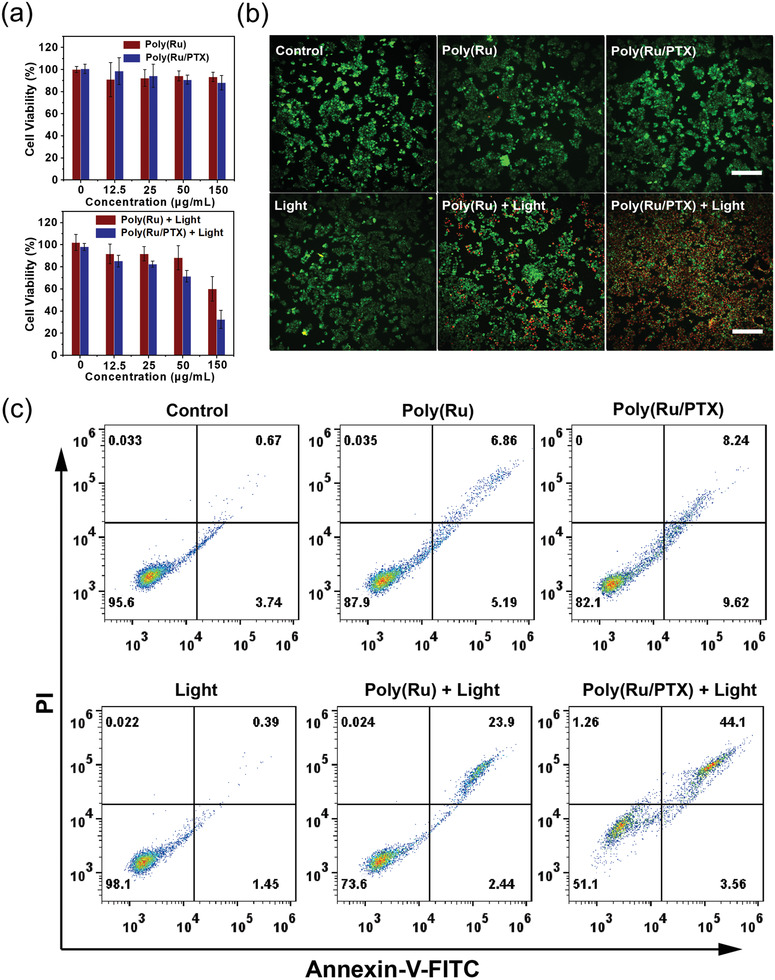
In vitro cytotoxicity of Poly(Ru/PTX) nanoparticles. a) Cell viability of MCF‐7 cells incubated with the nanoparticles at various concentrations with (bottom) or without (top) laser irradiations. b) Calcein AM (green) and propidium iodide (red) co‐staining fluorescence imaging of MCF‐7 cells after different treatments. Scale bar: 100 µm. c) Apoptosis and necrosis analyses using flow cytometry toward MCF‐7 cells after different treatments. Red light irradiation (671 nm, 200 mW cm^−2^, 15 min) was conducted after cells were incubated with different nanoparticles.

Encouraged by the excellent in vitro properties, we then studied the potential of Poly(Ru/PTX) nanoparticles to be used for cancer treatment **(** **Figure** **5a**. In the absence of red light irradiation, no significant cytotoxicity was observed from Poly(Ru) and Poly(Ru/PTX) nanoparticles, indicating their good biocompatibility. In contrast, after 15 min of red light irradiation, cell viability in both groups significantly decreased in a concentration‐dependent manner. Additionally, phototherapy mediated with Poly(Ru/PTX) nanoparticles showed a higher cytotoxicity than phototherapy mediated with Poly(Ru) nanoparticles. The cell viability of cancer cells treated with Poly(Ru/PTX) nanoparticles and red light was 32.4%, which was 1.8‐fold lower than cells in the Poly(Ru)‐nanoparticle‐mediated phototherapy group. The significantly enhanced therapeutic effect observed in the Poly(Ru/PTX) nanoparticles was attributed to the combined effect of the PDT and ROS‐triggered drug release.

Live–dead cell staining was performed by using a calcein acetoxymethyl ester (calcein AM) and propidium iodide (PI) assay to visualize the phototherapy effect of Poly(Ru/PTX) nanoparticles (Figure 5b). In the control and light groups, the cells emitted strong green fluorescence, suggesting that sole red light irradiation did not cause damage to cells. As expected, after 671 nm laser irradiation (200 mW cm^−2^, 15 min), Poly(Ru/PTX) nanoparticles induced much more cell death than that of Poly(Ru) nanoparticles as shown by stronger red fluorescence, which was consistent with the methylthiazolyldiphenyl‐tetrazolium bromide (MTT) assay results. Significantly, most cells treated with Poly(Ru/PTX) nanoparticles were alive with strong green fluorescence, which was similar to the control and light group. This indicates that the Poly(Ru/PTX) nanoparticles have good biocompatibility while maintaining the desired cytotoxicity when activated by red light.

We further evaluated the cell apoptosis induced by Poly(Ru/PTX) nanoparticles using an Annexin V‐ fluorescein isothiocyanate (FITC) apoptosis detection kit (Figure 5c). The apoptotic ratios of Poly(Ru) and Poly(Ru/PTX) nanoparticles treated with red light were much higher than the groups without red light irradiation, suggesting that red light could significantly enhance the anticancer effects of the nanoparticles. Notably, the cells exposed to Poly(Ru/PTX) nanoparticles and red light irradiation exhibited the highest apoptotic ratio (47.7%) than any other groups. These results agreed with those from in vitro cytotoxicity assay, suggesting that chemo‐photodynamic therapy with Poly(Ru/PTX) nanoparticles is more efficient in inducing tumor cell death, which exhibits great potential for in vivo applications.

### 2.4 In Vivo Antitumor Efficacy

**Figure 6:**
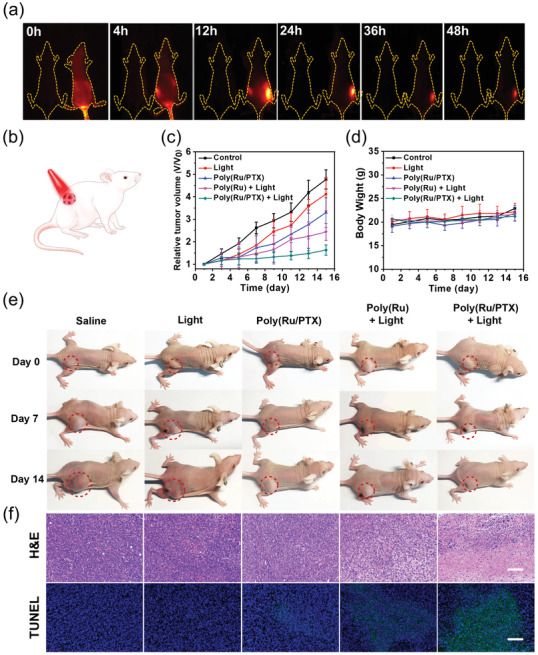
In vivo antitumor efficacy in 4T1 tumor‐bearing mice. a) Fluorescence images of 4T1 tumor‐bearing mice after intravenous injection of PBS (left, control) and dye‐loaded Poly(Ru/PTX) nanoparticles (right). Images were taken at 0, 6, 12, 24, and 36 h after injection. b) A schematic illustration shows red light irradiation on a mouse model. c) Relative tumor volume changes of tumor‐bearing mice during different treatments for 14 days. d) The body weight changes of mice in different groups. e) Photographs of representative mice during different treatments. f) Ex vivo histological analyses of tumor sections after different treatments by H&E and TUNEL staining. Scale bar: 100 µm.

To determine the optimal in vivo phototherapeutic time point, we investigated the accumulation of nanoparticles in the subcutaneous 4T1 xenograft tumor mouse model using in vivo fluorescence imaging (**Figure** **6**a). After injecting dye‐loaded Poly(Ru/PTX) nanoparticles into the tail vein of live tumor‐bearing mice, clear fluorescence signals were observed in the tumor regions at 12 h postinjection time, suggesting that 12 h postinjection might be the best time for light irradiation (Figure S36, Supporting Information). Although the fluorescence gradually decreased after 12 h due to metabolism, even after 48 h bright fluorescence could be still observed. Conversely, fluorescence signals were not detected at the tumor sites for the control group, which further confirmed that the fluorescence was due to the injected nanoparticles. Thus, Poly(Ru/PTX) nanoparticles could effectively accumulate at the tumor site due to the prolonged blood circulation and the passive tumor‐targeting effect of nanoparticles (Figure S37, Supporting Information).

The in vivo antitumor activity was further investigated by intravenously injecting Poly(Ru/PTX) nanoparticles into 4T1 tumor‐bearing mice, followed by local activation with 671 nm light (200 mW cm^−2^, 30 min) 12 h after injection (Poly(Ru/PTX) + Light) (Figure 6b). This irradiation dose used in in vivo can efficiently cause photocleavage of Poly(Ru/PTX) (Figure S38, Supporting Information). Four control experiments were also conducted as follows: 1) the mice were injected with phosphate buffered saline (PBS) (control group); 2) the mice were irradiated (light group); 3) the mice were injected with Poly(Ru) nanoparticles and irradiated (Poly(Ru) + Light group); and 4) the mice were injected with Poly(Ru/PTX) nanoparticles and not irradiated (Poly(Ru/PTX) group). The tumor volumes of each group were monitored for 14 days to evaluate their in vivo therapeutic efficacy (Figure 6c,e). The tumor volume of the control and light‐only treatment groups exhibited rapid growth within 14 days. As expected, mice in both Poly(Ru/PTX) and Poly(Ru) + Light groups slowed tumor growth. In particular, Poly(Ru/PTX) + Light demonstrated the most efficient anticancer effect with ≈65% tumor growth inhibition. The tumor weight of the excised tumor also supported the results that the combined chem‐photodynamic therapy efficiently inhibitated the tumor growth (Figure S39, Supporting Information).

To further evaluate the in vivo antitumor activity, histological analysis was performed after the 14 day treatment. Tumor tissues from 4T1 tumor‐bearing mice were collected for the hematoxylin and eosin (H&E) and terminal deoxynucleotidyl transferase mediated UTP end labeling (TUNEL) staining (Figure 6f). The results of the H&E staining of tumor tissues showed significant tumor cell nuclear ablation in the Poly(Ru/PTX) + Light group, which can be attributed to the Ru‐induced ROS generation and subsequent release of PTX. Similarly, TUNEL staining also revealed that the highest level of apoptosis in tumor tissues was obtained from mice in the Poly(Ru/PTX) + Light group, while tumors in the other groups exhibited weak green fluorescence, implying only slight apoptosis. Overall, these results confirm the excellent tumor accumulation and anticancer activity of Poly(Ru/PTX) nanoparticles under red light irradiation.

Since nanomedicines are to be used in vivo, it is paramount to perform a rigorous examination of the therapy's biosafety. According to our design, the Poly(Ru/PTX) nanoparticles could stably hold the therapeutic agents during blood circulation, minimize drug leakage, and reduce systemic toxicity. The biosafety of Poly(Ru/PTX) nanoparticles was investigated by observing the general locomotor activity, monitoring body weights, and analyzing the H&E staining of major organs in mice. For the duration of the treatment period, there were no obvious weight changes or abnormal behaviors in the mice (Figure 6d). No observable hemorrhage, inflammatory cell infiltration, or change to physiological morphology was found in the main organs (heart, liver, lung, spleen, and kidney) in all treated groups (Figure S40, Supporting Information). To evaluate the long‐term safety of Poly(Ru/PTX), the nondegradable polyacrylate‐based analog (MPEG‐*b*‐PAPH‐(Ru/PTX)), was also synthesized as a comparison polymer via reversible‐addition–fragmentation chain transfer (RAFT) polymerization, and the identical deprotection and click reactions (Figure S2, Supporting Information). All other chemical structures were unchanged except for the nondegradable polyacrylate backbone (Figures S21–S24, Supporting Information). The same dose of Poly(Ru/PTX) and MPEG‐*b*‐PAPH‐(Ru/PTX) nanoparticles were subcutaneously injected into the mice and kept for 30 days. The H&E staining of skin tissue showed that MPEG‐*b*‐PAPH‐(Ru/PTX) nanoparticles caused a high level of neutrophilic infiltration into the mice, but the mice in the Poly(Ru/PTX) nanoparticles and PBS groups experienced much less (Figure S41, Supporting Information). These results can be attributed to the promising biocompatibility and degradability of polycarbonate‐based Poly(Ru/PTX). Hemolysis analysis further confirmed that Poly(Ru/PTX) nanoparticles have good blood compatibility (Figure S42, Supporting Information). Together, the above results have fully demonstrated the excellent biosafety of the nanoparticles in vivo.

## 3 Conclusion

We have synthesized a red‐light‐activatable metallopolymer, Poly(Ru/PTX), to optimize chemo‐photodynamic therapy. The Ru complex and PTX were covalently grafted to the biodegradable polymer backbone, resulting in the formation of an ROS‐sensitive linkage between the therapeutic agents and the backbone. The high stability of the polymeric nanoparticles reduced drug leakage, while both therapeutic agents were delivered to the tumor site in an “inactivated” state. Red light irradiation at the tumor induced the cleavage of the Ru complexes and the generation of ^1^O_2_, which sequentially cut the linkage and released free PTX. Thus, Poly(Ru/PTX) nanoparticles demonstrated a synergistic chemo‐photodynamic therapy, which was demonstrated both in vitro and in vivo. To the best of our knowledge, this study presents the first metallopolymer with a sequential dual‐model strategy for the on‐demand release of photosensitizers and anticancer drugs in cancer treatment.

## 4 Experimental Section

The experimental section is available in the Supporting Information.

## Conflict of Interest

The authors declare no conflict of interest.

## Supporting information

Supporting Information

## Data Availability

Research data are not shared.
