# Supplementary material for: A Sequential Dual‐Model Strategy Based on Photoactivatable Metallopolymer for On‐Demand Release of Photosensitizers and Anticancer Drugs
Source: Adv Sci (Weinh). 2021 Oct 18;8(23):2103334. doi: 10.1002/advs.202103334 (PMC8655221; doi:10.1002/advs.202103334)
Supplement: Supplementary file 1 — Supporting Information [file ADVS-8-2103334-s001.pdf]

© 2021 Wiley-VCH GmbH

ADVANCED SCIENCE
Open Access

Supporting Information

for *Adv. Sci.,* DOI: 10.1002/advs.202103334

A Sequential Dual-model Strategy based on Photoactivatable Metallopolymer for On-Demand Release of Photosensitizers and Anticancer Drugs

Maomao He, Guangli He, Peiyuan Wang, Suhua Jiang, Ziyue Jiao, Dongmei Xi, Pengcheng Miao, Xuefei Leng, Zhiyong Wei, Yang Li*, Yanjun Yang, Ran Wang, Jianjun Du, Jiangli Fan, Wen Sun*, and Xiaojun Peng*

Supporting Information

A Sequential Dual-Model Strategy based on Photoactivatable Metallopolymers for On-Demand Release of Photosensitizers and Anticancer Drugs

Maomao He, Guangli He, Peiyuan Wang, Suhua Jiang, Ziyue Jiao, Dongmei Xi, Pengcheng Miao, Xuefei Leng, Zhiyong Wei, Yang Li*, Yanjun Yang, Ran Wang, Jianjun Du, Jiangli Fan, Wen Sun*, and Xiaojun Peng*

1. Materials

$RuCl_3$•$3H_2O$ (99.9%), 2,2',6',2''-terpyridine (99%), 2,2'-biquinoline (98%), 1,6-dihydroxyhexane (99%), 4-hydroxybenzonitrile (98%), paclitaxel (PTX) (97%), propiolic acid (95%), 1,1'-carbonyldiimidazole (CDI) (97%), and N-Boc-4-piperidinol (98%) were purchased from Sigma-Aldrich. Trimethylolpropane (98%), 1,8-Diazabicyclo [5.4.0] undec-7-ene (DBU) (99%), and Polyethylene glycol monomethyl ether (MPEG, M_n = 5.0 kg/mol) were purchased from Aladdin (Shanghai, China). All general chemicals for fluorescence detection including 1,3-diphenylisobenzofuran (DPBF) and 3-(4,5-dimethyl-2-thiazolyl)-2,5-diphenyl-2-H-tetrazolium bromide (MTT) were purchased from Energy Chemical Co. Silica gel (200-300 mesh) used for column chromatography was purchased from Qingdao Ocean Chemicals. Calcein-AM/propidium iodide (PI) detection kit was purchased from Beyotime Biotechnology Co. (China). Annexin VFITC/propidium iodide (PI) Apoptosis Detection Kit was purchased from KeyGEN BioTECH Ltd. All other reagents were commercially available and used as received, unless noted otherwise. The 1-(3,5-Bis

(trifluoromethyl)-phenyl-3-cyclohexyl-2-thuourea) (TU) was synthesized according to previously reported procedure.[1] Human breast cancer MCF-7 cells and mouse breast cancer 4T1 cells were purchased from Institute of Basic Medical Sciences (IBMS) of the Chinese Academy of Medical Sciences.

2. Methods

^{1}H-NMR and ^{13}C-NMR spectra were performed with Bruker Avance Ⅲ 500 spectrometer. Mass spectrometric (MS) data was carried out using LTQ Orbit rap XL instruments. Absorption and emission spectra were performed with a UV-Vis spectrophotometer (Lambda 750S) and a VAEIAN CARY Eclipse fluorescence spectrophotometer (Serial No. FL0812-M018), respectively. The weight average molecular weight and distribution were determined using Waters 1515 HPLC system with tetrahydrofuran (THF) as the eluent. Transmission electron microscopy (TEM) images were measured on HT7700 EXALENS. The diameter of the nanoparticles was determined by dynamic light scattering (DLS) on Malvern Zetasizer Nano ZS90 (Malvern, UK). A laser λ = 671 nm was employed as the light source for *in vitro* and *in vivo* experiments. The output power of the laser was controlled by a fiber coupled laser system (FC-671-1W, Changchun New Industries Optoelectronics Technology) and measured by a power meter (LP100/TS15, Changchun New Industries Optoelectronics Technology). Confocal laser scanning microscope (CLSM) images were performed on Olympus FV3000 confocal laser scanning microscope.

3. Synthesis

3.1 Synthesis of Poly(Ru/PTX) and Poly(Ru)

Synthesis of TMP carbonate imidazole carboxylate (TMPIC)

Trimethylolpropane (TMP) (5 g, 37 mmol) was dissolved in 100 mL acetone by stirring at room temperature for 10 min. The flask was flowed by constant N_2, using a long needle, and equipped with a powder funnel on top of the needle. Then, 1,1’-carbonyldiimidazole (CDI) (14 g, 86 mmol) was slowly added over a period of 1

h at room temperature with high stirring. The next portion was added after the complete dissolution of the previous addition. After that, a white cloudy solution was obtained with a further stirring at room temperature for 1 h. The stirring was stopped and the mixture was keep for 1 h. White precipitate formed, which was collected by filtration, washed with diethyl ether and dried under vacuum to afford a fluffy white powder (5.46 g, 58%).

Synthesis of propargyl-TMP-carbonate (TMCP-Boc)

TMPIC (1.00 g, 5.74 mmol), N-Boc-4-piperidinol (1.73 g, 8.61 mmol) and CsF (18 mg, 0.11 mmol) were added into a round-bottom flask with flame and argon purge. Subsequently, 25 mL acetone was added under argon and further vortexed for 24 h at 20 °C. The insoluble substance in the reaction system was removed by filtration. The filtrate was collected, concentrated and subjected to column chromatography (dichloromethane/ethyl acetate = 2/1) to obtain a colorless liquid, which was further purified by crystallization from n-hexane at -20 °C to give clean monomer as a white crystal (1.48 g, 67 %).

Synthesis of methoxy polyethyleneglycol-polycarbonate (MPEG-*b*-PTMCP-Boc)

In a nitrogen-filled glovebox, a 50 ml round-bottom flask equipped with a magnetic stir bar was charged with MPEG (0.601 g, 0.12 mmol), TMCP-Boc (1.16 g, 3.0 mmol), and TU (55 mg, 0.15 mmol). 6 ml THF (0.5 M) was then added and the reaction mixture was stirred until all of the chemicals completely dissolved. Next, DBU (15 μL, 0.10 mmol) was added and the reaction mixture was further stirred at 30℃ for 24 h. Afterwards, the mixture was precipitated in diethyl ether and the solid was collected following centrifugation and decanting the supernatant. This process was repeated twice to obtain the desired product as a white solid (1.44 g, 82 %).

Deprotection of MPEG-*b*-PTMCP-Boc

MPEG-*b*-PTMCP-Boc (0.5 g, 0.05 mmol) was dissolved in mixed solvent of 5 mL dichloromethane (DCM) and 5 mL TFA at -10 °C under Ar protection. The solution was further stirred at 25 °C for 1 h and concentrated by vacuum rotary evaporation.

The product was redissolved in 5 mL deionized water, then the solution was dialyzed (MW cutoff, 1000 Da) in deionized water for 48 h and lyophilized to afford a white powder (0.38 g, 91%).

Synthesis of 4-[(6-hydroxyhexyl) oxy] benzonitrile (Hob)

4-Hydroxybenzonitrile (7.14 g, 60 mmol), K_2CO_3 (8.28 g, 60 mmol) and 0.05 g KI were added into 70 mL ethanol. Then, 20 mL ethanol containing 6-chlorohexanol (10 mL, 0.07 mol) was added dropwise before the mixture was refluxed for 24 h under Ar atmosphere. The crude product was purified by column chromatography (n-hexane/ethyl acetate = 1/1) to yield a white waxy solid (10.91 g, 89%).

Synthesis of 6-(4-cyanophenoxy) hexyl alkynoate (CHA)

4-((6-hydroxyhexyl)oxy) benzonitrile (0.43 g, 2 mmol) and propionic acid (0.14 g, 2 mmol) were dissolved in 5 mL DCM and the mixture was cooled down to 0 °C. Then the solution of DCC (0.42 g, 2 mmol) and DMAP (0.024 g, 0.2 mmol) dissolved in 5ml DCM was added dropwise and stirred overnight. The precipitate was filtered, and then the solvent was removed by rotary evaporation. The yellow oil was purified by column chromatography (n-hexane/ethyl acetate = 50/1 to 5/1) to give colorless crystals (0.54 g, 84%).

Synthesis of $Ru(tpy)Cl_3$

$RuCl_3 \cdot 3H_2O$ (0.52 g, 2.0 mmol) and 2,2′;6′,2″-terpyridine (tpy, 0.56 g, 2.0 mmol) were dissolved in 150 mL ethanol. Then, the mixture was heated to 85 °C for 4 h under vigorous stirring. After that, the solution was cooled down to room temperature. The brown powder was collected by filtration washed with ethanol and diethyl ether, and then air-dried to obtain brown powder (0.70 g, 80%).

Synthesis of [Ru(tpy)(biq)(Cl)]Cl

$Ru(tpy)Cl_3$ (0.35 g, 0.8 mmol) and 2,2’-biquinoline (biq, 0.2 g, 0.8 mmol) were mixed in 2:1 ethanol/water mixture (50 mL) and the solution was bubbled with Ar for 10 min. The reaction mixture was refluxed in the dark under Ar for 24 hours. After that, the mixture was evaporated under reduced pressure and purified by column

chromatography with silica gel (eluent: DCM/methanol = 5:1), to obtain the product as a violet powder (0.48 g, 61%).

Synthesis of [Ru(tpy)(biq)(H_2O)](PF_6)$_2$

[Ru(tpy)(biq)(Cl)]Cl (0.12 g, 0.18 mmol) and $AgPF_6$ (0.10 g, 0.40 mmol) were dissolved in 1:1 methanol/water mixture (10 mL). The solution was degassed and stirred under Ar at 30 °C for 2 h. The solution was cooled and filtered to remove AgCl. The solvent of the reaction was reduced to 3 mL. Then, an aqueous solution of KPF_6 was added. The precipitate was filtered, washed with water, and lyophilized to give a purple solid (0.11 g, 72%).

Synthesis of [Ru(tpy)(biq)(CHP)](PF_6)$_2$ (Ru-A)

Generally, [Ru(tpy)(biq)(H_2O)](PF_6)$_2$ (80 mg, 0.084 mmol) and CHA (40 mg, 0.147 mmol) were mixed in 10 mL acetone. The solution was degassed for 10 min and stirred for 2 h at room temperature in the dark. After that, the solvent was removed by rotary evaporation under reduced pressure. The crude product was purified by silica gel column chromatography (DCM/methanol = 10/1) to obtain a red solid (78 mg, 79%).

Synthesis of Alkynyl-Paclitaxel (PTX-A)

PTX-A was synthesized by the esterification of PTX with propiolic acid. Palitaxel (300 mg, 0.35 mmol) and propiolic acid (36.9 mg, 0.52 mmol) were dissolved in dry 10 mL DCM under a Ar atmosphere, and the solution was cooled down in an ice-water bath. Then dicyclohexylcarbodiimide (DCC) (73.2 mg, 0.35 mmol) and 4-dimethylaminopyridine (4-DMAP) (1.2 mg, 0.01 mmol) were dissolved in 5 mL DCM and added dropwise to the solution over 30 min. After 1 h, the reaction mixture was moved to room temperature and stirred continuously overnight. The solution was filtered, concentrated, and separated on a silica column chromatography (n-hexane/ethyl acetate = 2/1) to obtain a white solid (0.73 g, 72%).

Synthesis of Poly(Ru/PTX)

The amphiphilic Poly(Ru/PTX) copolymer were synthesized by amino-alkynoate

click reaction. In an Ar atmosphere glovebox, MPEG-*b*-PTMCP (100 mg, 0.127 mmol free amino groups) were dissolved in 2 mL dry dimethylformamide (DMF), PTX-A (50 mg, 0.06 mmol) and Ru-A (60 mg, 0.06 mmol) were added to the reaction vial and stirred overnight at room temperature in the dark. At the end of reaction, the solution was dialyzed (MW cutoff, 3500 Da) against deionized water for 48 h to remove organic solvents. The dialysate was lyophilized to give a red solid (174 mg, 83%).

Poly(Ru) copolymer without PTX, as a comparison, was synthesized by a similar procedure. The synthetic route was shown in Figure S1 (Figure S18).

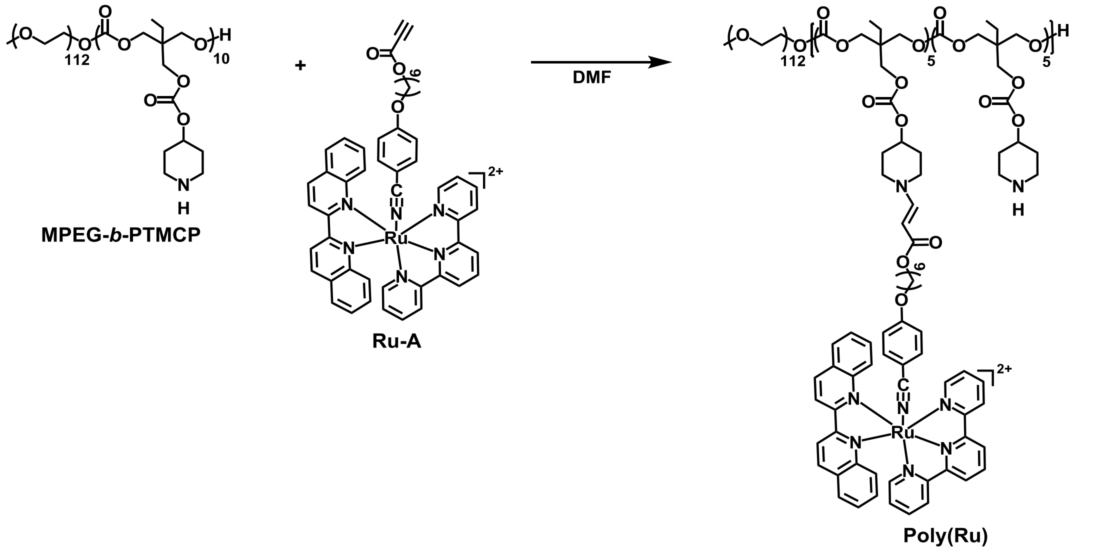

Figure S1. Synthetic route of PTX-free control polymer Poly(Ru).

3.2 Synthesis of MPEG-*b*-PAPH-(Ru/PTX)

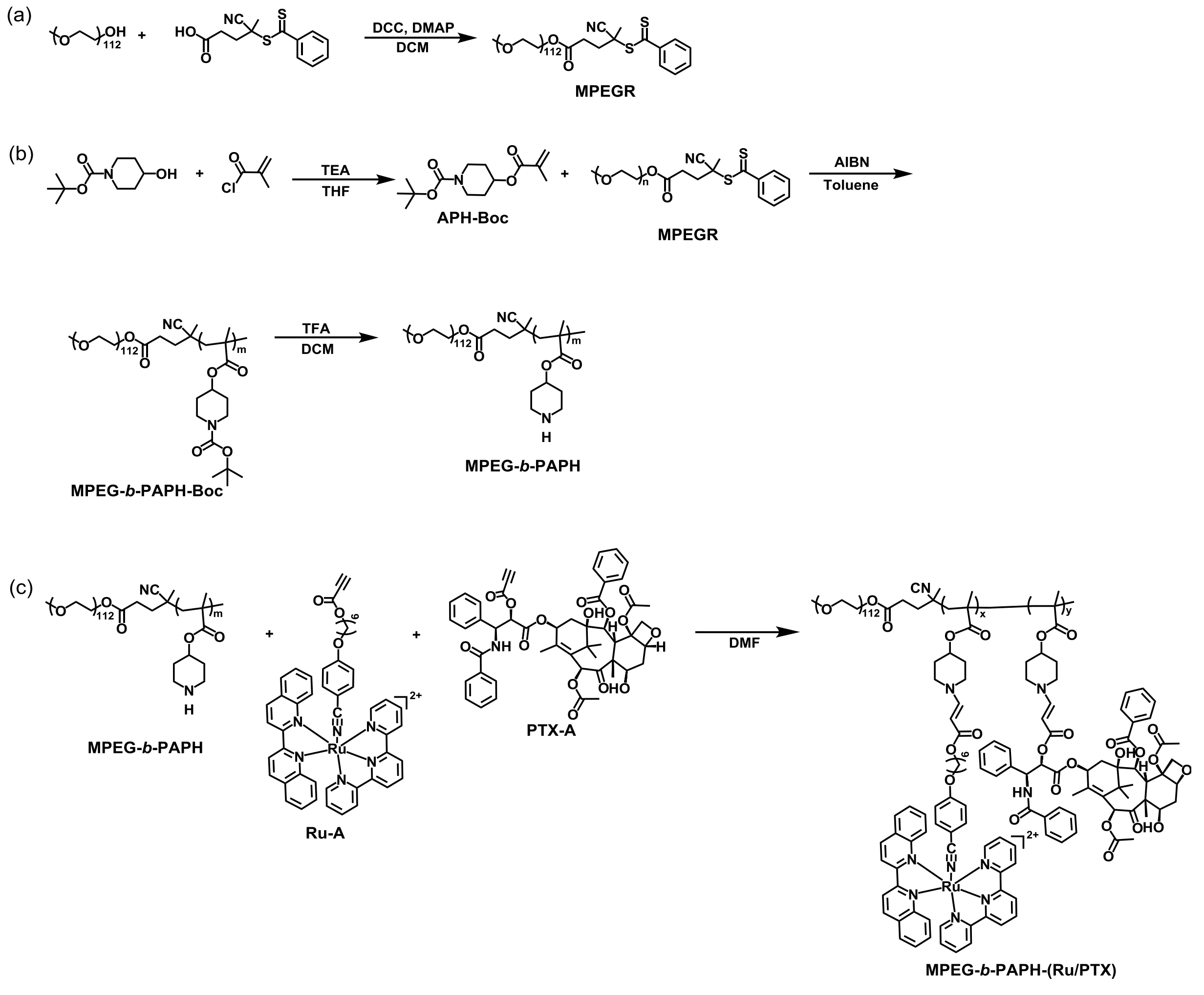

Figure S2. Synthetic route of MPEG-*b*-PAPH-(Ru/PTX). The synthesis of a) macromolecular chain-transfer agent MPEGR; b) nonbiodegradable polymer backbone MPEG-*b*-PAPH and c) MPEG-*b*-PAPH-(Ru/PTX).

Synthesis of MPEG macromolecular chain transfer agent (MPEGR)

A solution of DCC (0.125 g, 5.94 mmol) in 5 mL DCM was added dropwise to a 20 mL DCM solution of 4-cyano-4-(phenylcarbonothioylthio) pentanoic acid (0.168 g, 0.60 mmol), MPEG (2.43g, 0.48 mmol), and DMAP (6.1 mg, 0.05 mmol) in an ice-water bath. After being filtrated, the filtrate was concentrated, and precipitated from diethyl ether. The solid was collected following centrifugation and decanting the supernatant. This process was repeated two times to obtain the desired product as a pink solid (2.26g, 86%).

Synthesis of Boc-piperidine-methacrylate (APH-Boc)

N-Boc-4-piperidinol (8.82 g, 43.8 mmol) and trimethylamine (4.87g, 48.18 mmol) were dissolved in 100 mL THF at 0 ℃. Then, the 150 mL THF solution of methacryloyl chloride (4.3 mL, 46.1 mmol) was added to the reaction mixture dropwise. Afterwards, the reaction mixture was stirred overnight at room temperature. The solvent was evaporated to get the crude product which was purified by silica gel column chromatography (n-hexane/ethyl acetate = 4/1) to obtain a white solid (6.36 g, 55%).

Synthesis of MPEG-*b*-PAPH-Boc

MPEGR (0.510 g, 0.1 mmol), APH-Boc (0.54 g, 2.0 mmol), and azodiisobutyronitrile (AIBN) (3.2 mg, 0.02 mmol) were dissolved in 5 mL toluene. The mixture was then degassed via three freeze-pump-thaw cycles and kept at 70 °C for 24 h. The solvent was evaporated to get the polymer, which was purified by precipitating from DCM in an excess of diethyl ether, and then dried under vacuum to obtain a pale red powder (0.84 g, yield: 81 %).

Synthesis of MPEG-*b*-PAPH-(Ru/PTX)

The MPEG-*b*-PAPH-Boc was deprotected and conjugated to Ru-A and PTX-A with the same procedure as Poly(Ru/PTX) described above.

4. Sample preparation

Preparation of Poly(Ru/PTX) nanoparticles

Poly(Ru/PTX) nanoparticles were prepared through adding water to an organic solution of the polymer. In particular, 1.8 mL deionized water was added dropwise into 0.2 mL THF containing 2 mg Poly(Ru/PTX) at a flow rate of about one drop per second and stirred in the dark for 30 min. The red solution was then dialyzed against deionized water for 48 h to remove organic solvents through a dialysis bag (MW cutoff, 3500 Da).

Drug release profiles of Poly(Ru/PTX) nanoparticles

In detail, Poly(Ru/PTX) nanoparticles solution was placed into a dialysis bag and then was immersed in the release medium (PBS, pH 6.8 and pH 7.4) before incubating in a continuous shaker at 37 °C. At predetermined time intervals, 1 mL of the medium was withdrawn and an equal volume of fresh release medium was refilled at various time points. To evaluate the photo-triggered release behavior, the sample solution were irradiated with 671 nm laser with a power intensity of 200 mW cm^{-2} for 15 min before measuring the release profiles. The samples without any laser illumination were used as the negative control group. The amount of Ru-H_2O and PTX released was measured using inductively coupled plasma mass spectrometry (ICP-MS) and high-performance liquid chromatography (HPLC), respectively.

Preparation of dye-loaded Poly(Ru/PTX) nanoparticles

Indocyanine Green (ICG) was encapsulated into the Poly(Ru/PTX) nanoparticles to examine its cellular uptake efficiency and *in vivo* biodistribution. 25 µL Dye solution (2 mg/mL in DMF) was added into 200 µL THF containing 2 mg Poly(Ru/PTX). The solution was stirred for 30 min at 300 rpm. After that, 1.8 mL deionized water was added dropwise into the solution at a flow rate of about one drop per second and another 30 min of stirring was continued. The solution was then dialyzed against deionized water for 48 h to remove organic solvents and un-encapsulated dye through a dialysis bag (MW cutoff, 3500 Da).

A similar procedure was followed for the preparation of Dye-loaded Poly(Ru)

nanoparticles.

5. Cell experiments

Cellular uptake

MCF-7 was seeded at a density of 1 x 10^5 cells/mL in 35 mm diameter μ-discs and cultured overnight in supplementary medium. The medium was replaced by fresh medium containing 50 μg/mL dye-loaded nanoparticles. The cells were incubated at different time periods. Subsequently, the nuclei were stained with Hoechst 33342 (1 μg/mL). The dye was further removed by washing three times with PBS. Images of live cells were taken using CLSM. Dye-loaded nanoparticles were excited with a 780 nm laser and detected in the range from 800 to 835 nm. The cell nuclei were excited with a 405 nm laser and detected in the range from 425 to 475 nm.

Intracellular ROS

The fluorescent probe DCFH-DA was used for detecting intracellular singlet oxygen ($^1O_2$) according to the manufacture instruction. Specifically, MCF-7 cells were incubated with medium containing 50 μg/mL with the nanoparticles for 6 h, and then incubated with DCFH-DA (1μg/mL) for another 30 min. After that, specific groups were irradiated by red light (671 nm, 200 mW cm^{-2}) for 15 min. Subsequently, the cells were washed with PBS again, followed by confocal fluorescence imaging (excited at 488 nm, monitored at 500-600 nm).

Fluorescence imaging of microtubules and microfilaments

MCF-7 cells were treated with different nanoparticles (equivalent to 1 μg/mL PTX) in medium for 6 h then irradiated for 15 min or in dark. After additional incubation for 24 h, the cells were fixed with 4% paraformaldehyde, and rinsed with PBS containing 0.1% Triton X-100. The cells were then incubated with the Tubulin-Tracker Red (diluted with immunofluorescence staining secondary antibody dilution buffer 1:100) for 1 h at 37 °C, rinsed with PBS containing 0.1% Triton X-100, stained with Hoechst 33342 and imaged through CLSM.

Flow cytometry experiment

Annexin V-FITC/PI Apoptosis Detection Kit was used for detection of nanoparticles-mediated cell apoptosis. Briefly, MCF-7 cells were seeded onto 35 mm confocal dishes for 12 h, then cells were treated with different nanoparticles in medium for 6 h then irradiated for 15 min or in dark. After incubation for 6 h, the cells were stained by Annexin V-FITC/PI Apoptosis Detection Kit according to the manufacture instruction, and finally the apoptosis effect in the cells was determined by analyzing 1×10^4 cells with flow cytometry.

6. In vivo experiments

Animals and tumor model

Female BALB/c nude mice, 6-week old, obtained from Laboratory Animal Center of Dalian Medical University. All the animal experiments involved in this work were approved by the Animal Care And Use Committee of Dalian University of Technology. Briefly, 1×10^6 4T1 cells were injected subcutaneously into the right flank region to establish the 4T1 tumor bearing mice. The tumor volume of 4T1 tumor-bearing mice was calculated as volume $A = b * c^2/2$ (a: length; b: width). After the tumor volumes were about 200 mm^3, mice were used for in vivo imaging and phototherapy.

***In vivo* fluorescence imaging**

Dye-loaded Poly(Ru/PTX) nanoparticles were used to evaluate the *in vivo* distribution in 4T1 tumor-bearing mice. 100 μL Dye-loaded Poly(Ru/PTX) nanoparticles was injected via the tail vein. Under general anesthetic, mice were optically imaged at different time points using an *in vivo* imaging system (IVIS Lumina imaging system) with the excitation wavelength at 808 nm.

***In vivo* antitumor evaluation**

The mice were divided into 5 groups (n = 5 per group) when the tumor reached 200 mm^3. The mice were treated via intravenous injection of (1) PBS (150 μL), (2) PBS (150 μL) with light irradiation, (3) Poly(Ru/PTX) nanoparticles (150 μL, 5 mg/kg), (4)

Poly(Ru) nanoparticles (150 μL, 5 mg/kg) with light irradiation, (5) Poly(Ru/PTX) nanoparticles with light irradiation. Light irradiation (671 nm laser, 200 mW cm^{-2}, 30 min) for group 4 and group 5 was carried out after 12 h intravenous injection of the nanoparticles. The treatment process was repeated three times on day 1, day 3, and day 5, respectively. The body weight and tumor volume of each mouse were measured every other day for 14 days. After the treatment, all mice were sacrificed, and the major organs and tumor tissues were collected for analysis.

Biosafety evaluation by hematoxylin and eosin (H&E) staining

The Poly(Ru/PTX) and MPEG-*b*-PAPH-(Ru/PTX) (with nonbiodegradable polymer backbone) nanoparticles solution (200 μL, 15 mg/kg) was injected subcutaneously into the thigh of mice. The mice were sacrificed on day 30 after administration. The skin tissues near the injection site was obtained by surgery for H&E staining to evaluate the inflammatory response of the mice.

Hemolysis Assay

The mouse blood was collected by orbital bleed into a microcentrifuge tube containing 20 μl of 10 % EDTA and 10 mL PBS and centrifugation at 1500 rpm for 15 min, then the plasma and buffy coat layer were removed. The red blood cells (RBCs) were collected and washed several times with PBS until red color cannot be seen in the upper solution. The obtained RBCs were suspended in 200 µL PBS, which were diluted by 9.8 ml PBS. After that, 0.5 mL RBCs suspension was added to 0.5 mL PBS containing different concentrations of Poly(Ru/PTX) nanoparticles to offer the final concentrations: 62.5, 125, 250, 500, and 1000 µg mL^{-1}. The 0.5 mL RBCs suspension incubated with 0.5 mL PBS or 0.5 mL water was used as negative control and positive control. The solution was gently mixed, left at 37 °C for 3 h. The red blood cells were collected via centrifugation at 10000 rpm for 3 min, and the absorbance value (576 nm) of the supernatant was measured by microplate reader. The hemolysis percentage was calculated according to the following formula:

Hemolysis (%) = [(Sample absorbance - Background absorbance) / (Positive control -

Negative control)] × 100%.

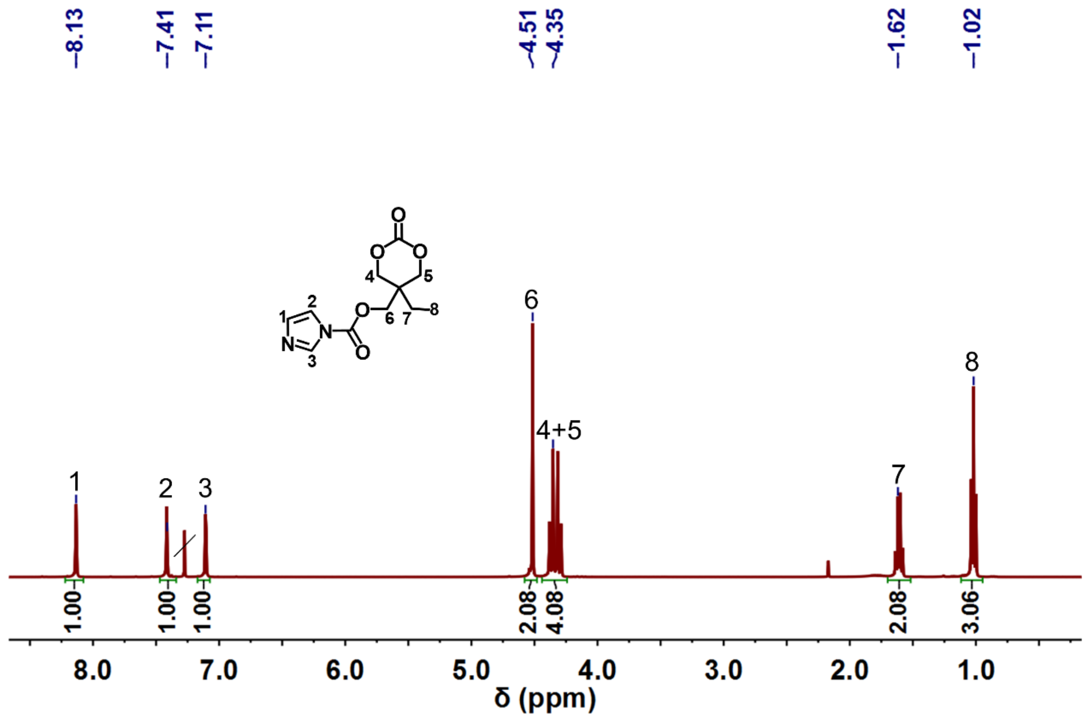

Figure S3. ^{1}H NMR spectrum of TMPIC (500 MHz, $CDCl_3$).

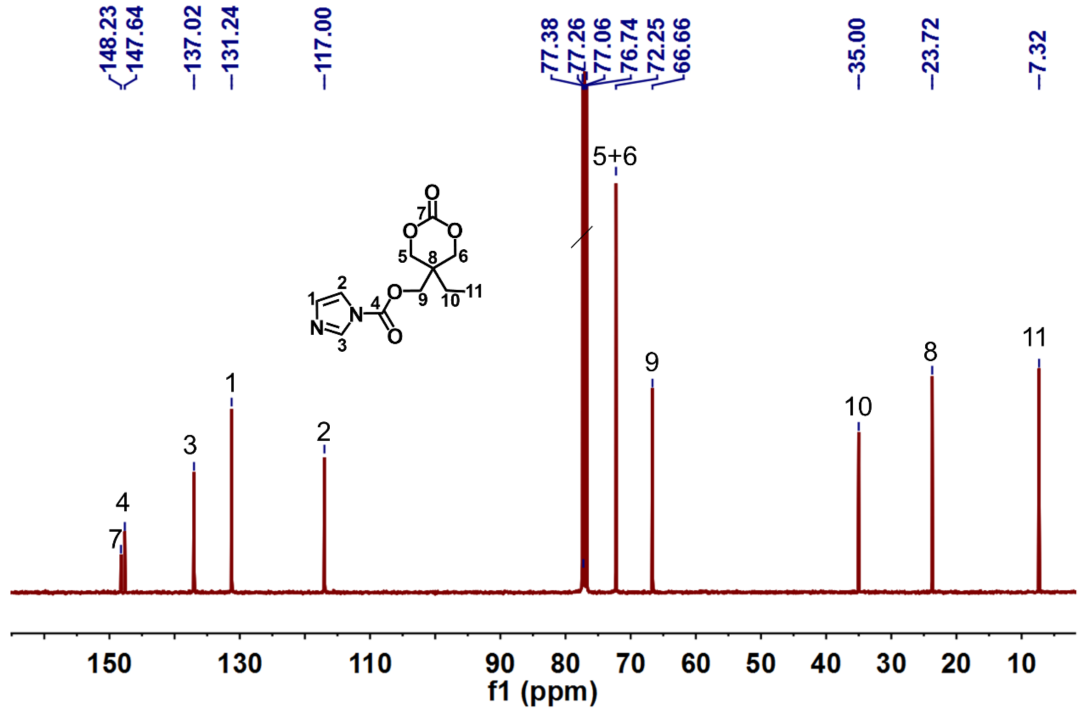

Figure S4. ^{13}C NMR spectrum of TMPIC (125 MHz, $CDCl_3$).

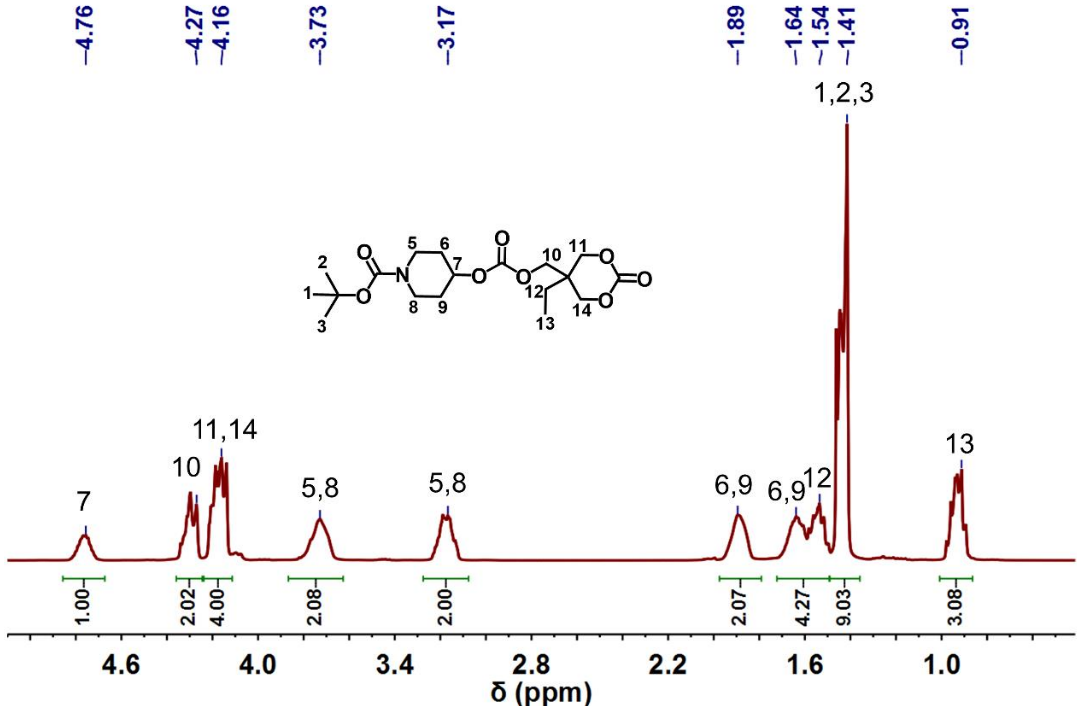

Figure S5. ^{1}H NMR spectrum of TMCP-Boc (500 MHz, $CDCl_3$).

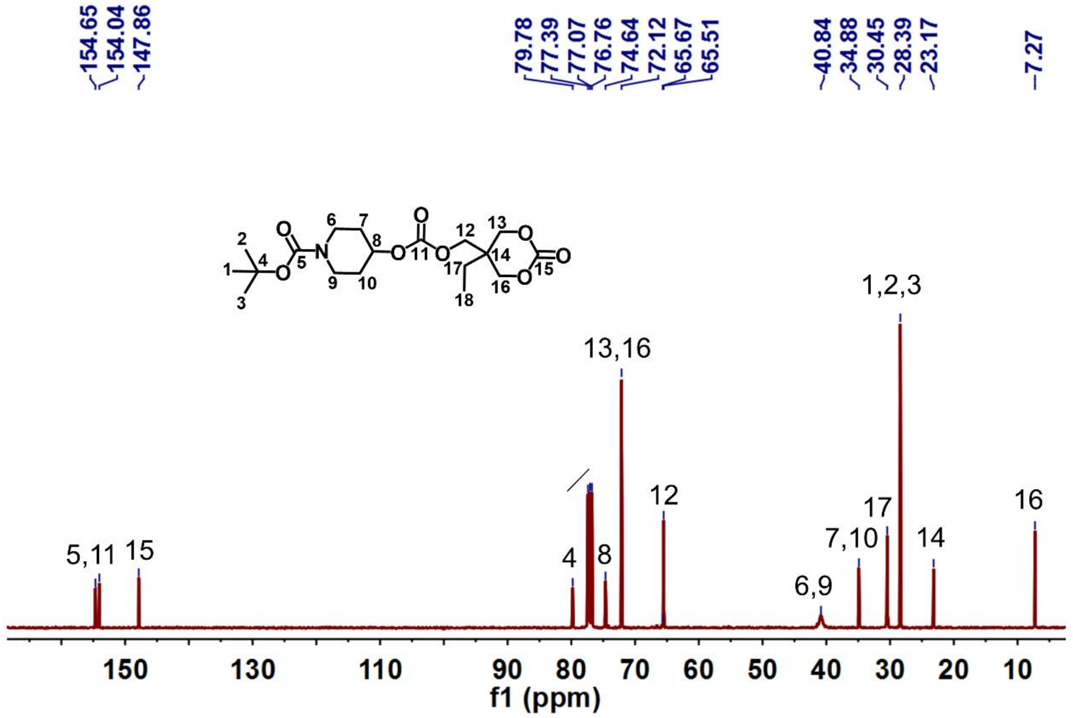

Figure S6. ^{13}C NMR spectrum of TMCP-Boc (125 MHz, $CDCl_3$).

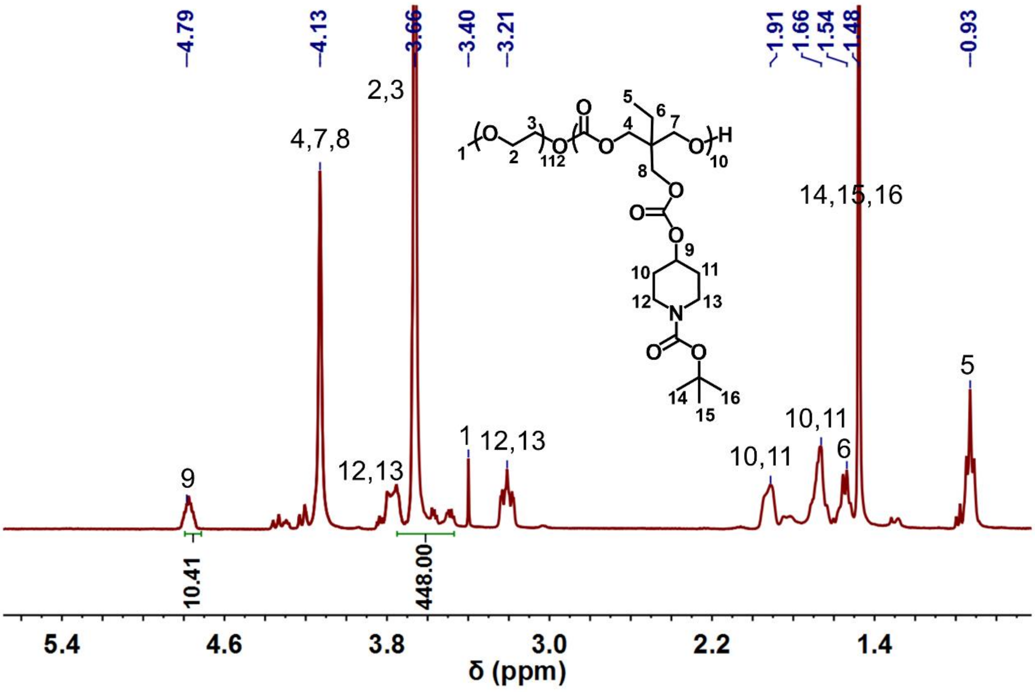

Figure S7. ^{1}H NMR spectrum of MPEG-*b*-PTMCP-Boc (500 MHz, $CDCl_3$).

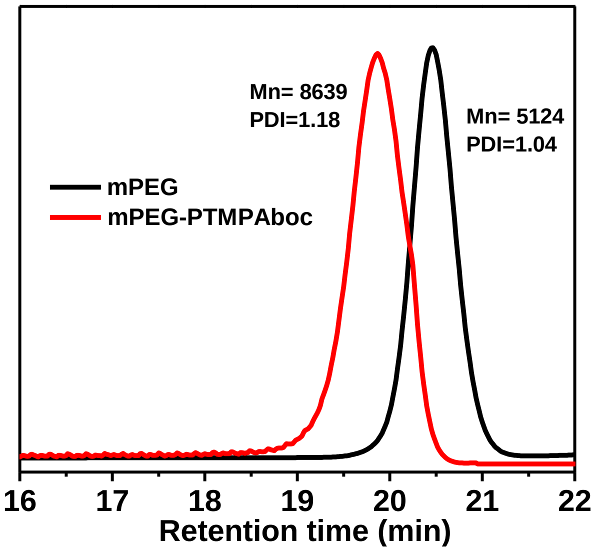

Figure S8. GPC traces for MPEG and MPEG-*b*-PTMCP-Boc. A clear peak shift to higher molecular weight of MPEG-*b*-PTMCP-Boc demonstrated the successful

polymerization.

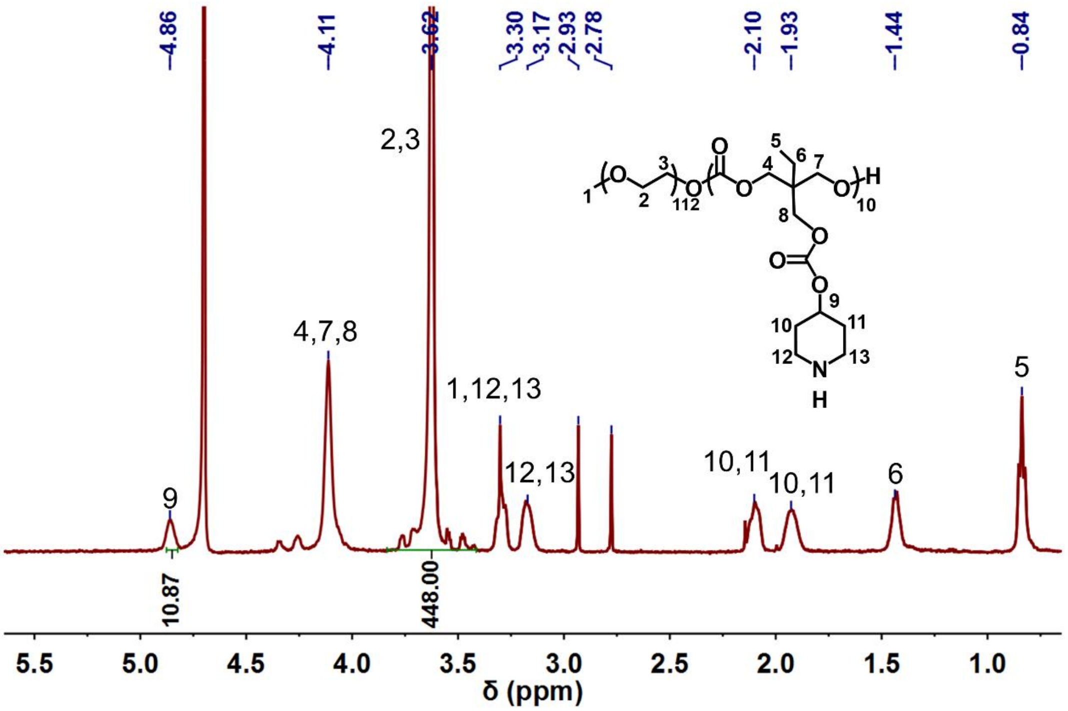

Figure S9. ^{1}H NMR spectrum of MPEG-*b*-PTMCP (500 MHz, D_2O).

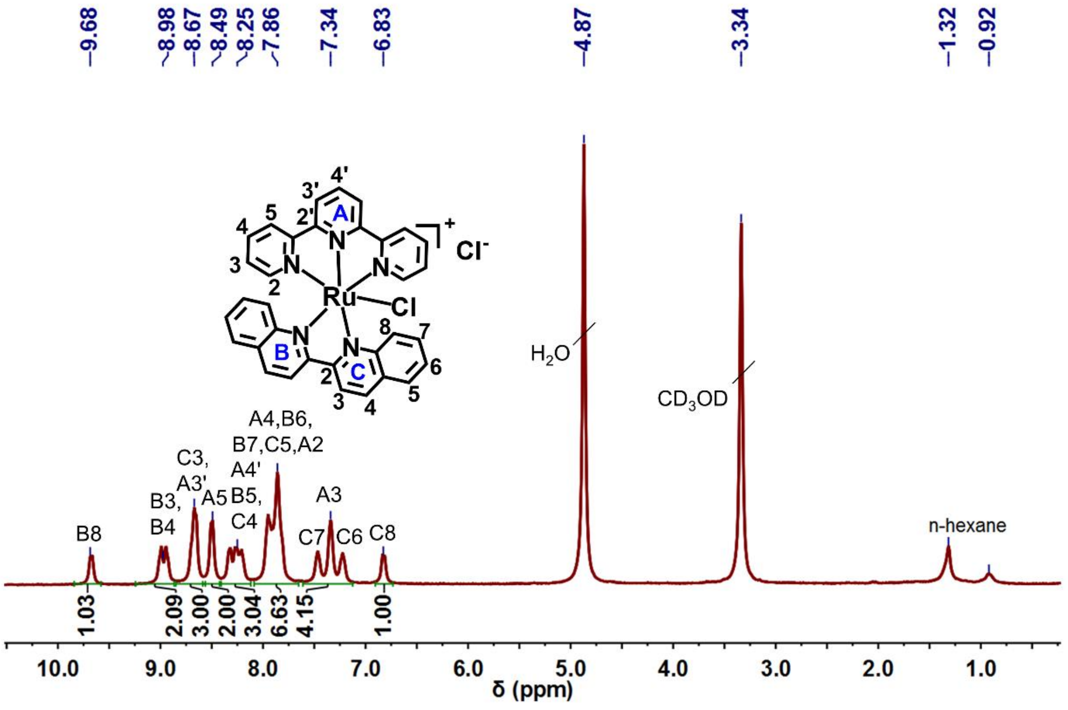

Figure S10. ^{1}H NMR spectrum of [Ru(tpy)(biq)Cl]Cl (500 MHz, CD_3OD).

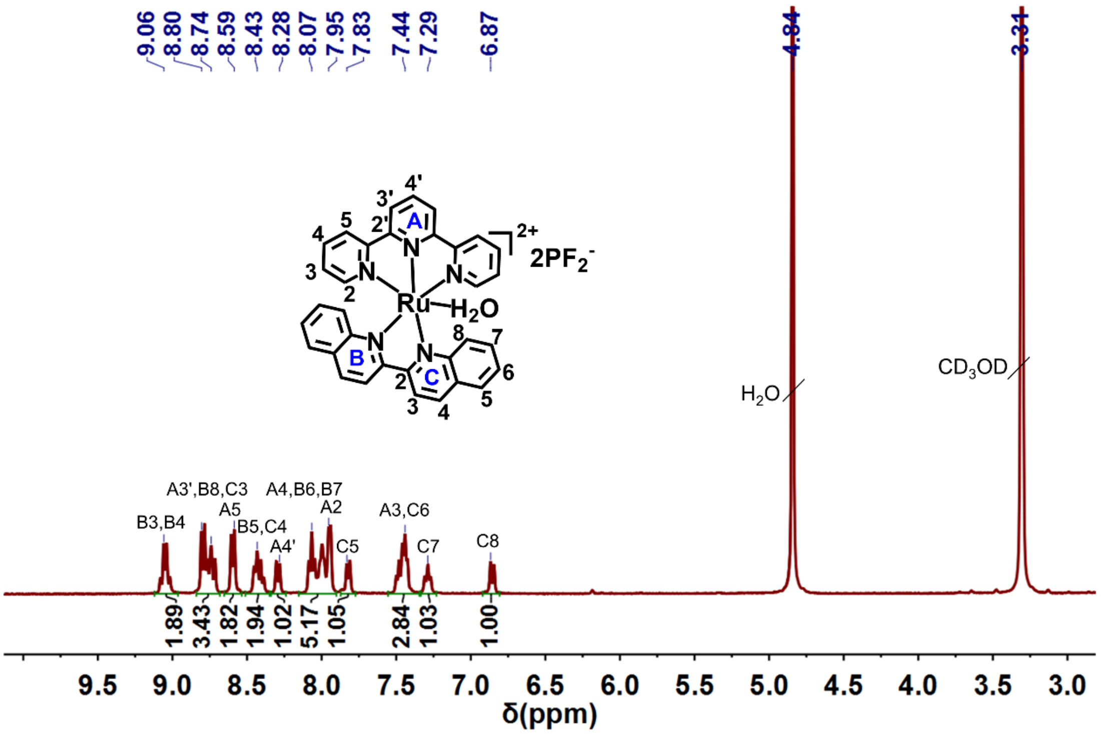

Figure S11. ^{1}H NMR spectrum of [Ru(tpy)(biq)(H_2O)]PF_6 (Ru-H_2O) (500 MHz, CD_3OD).

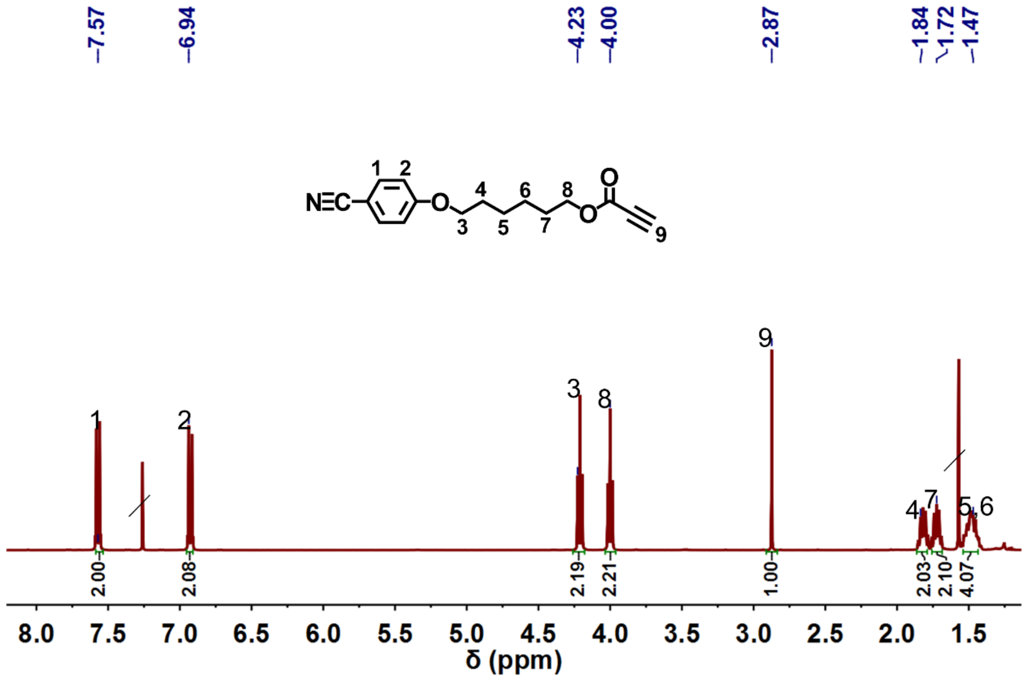

Figure S12. ^{1}H NMR spectrum of CHA (500 MHz, $CDCl_3$).

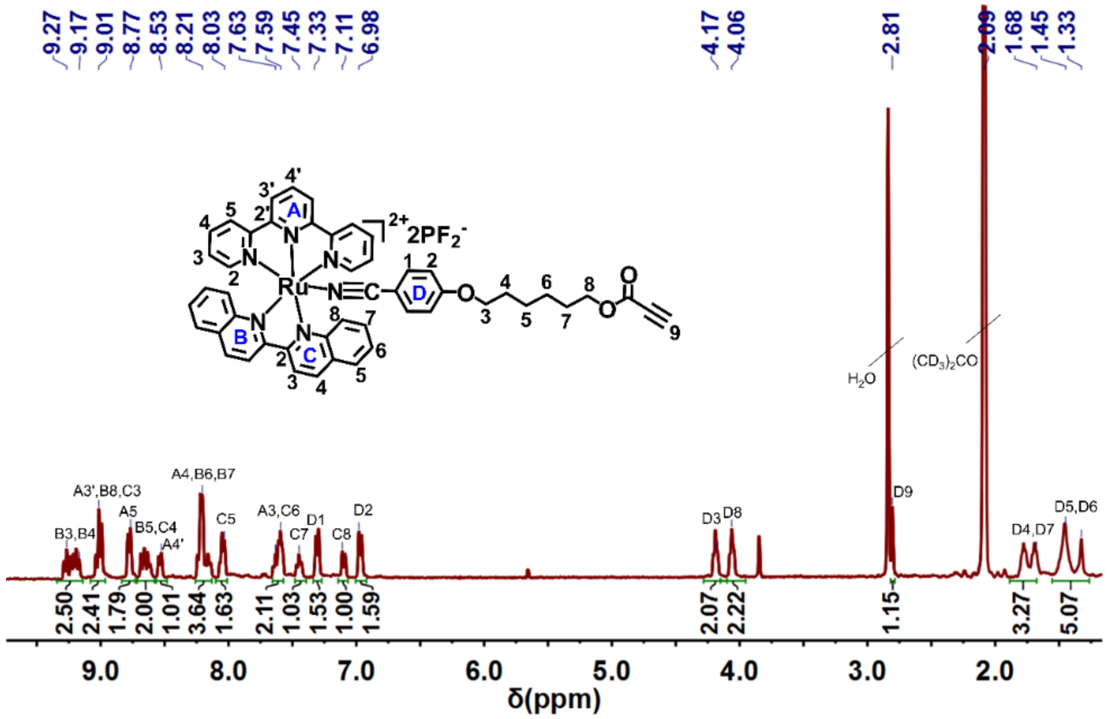

Figure S13. ^{1}H NMR spectrum of Ru-A (500 MHz, $(CD_3)_2CO$).

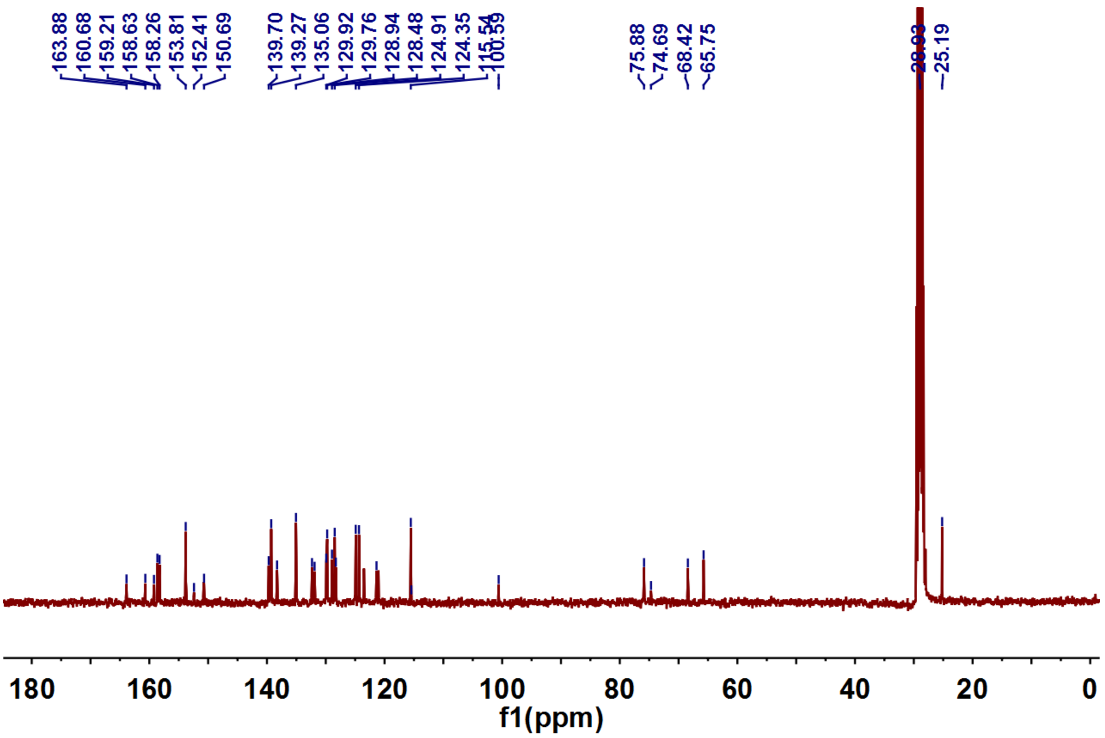

Figure S14. ^{13}C NMR spectrum of Ru-A (125 MHz, $(CD_3)_2CO$).

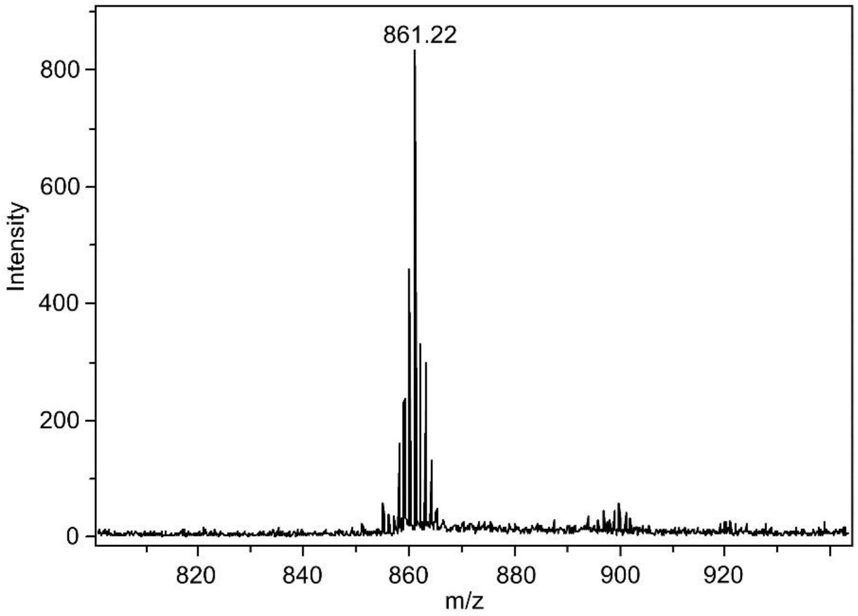

Figure S15. MALDI-TOF-MS spectrum of Ru-A.

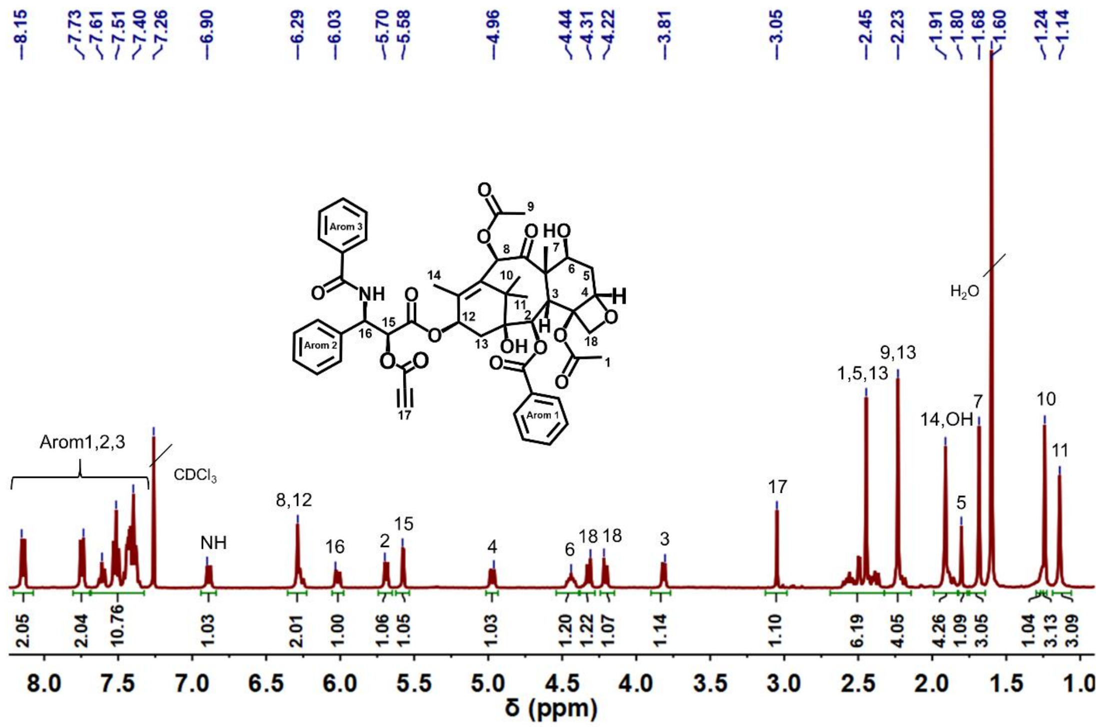

Figure S16. ^{1}H NMR spectrum of PTX-A (500 MHz, $CDCl_3$).

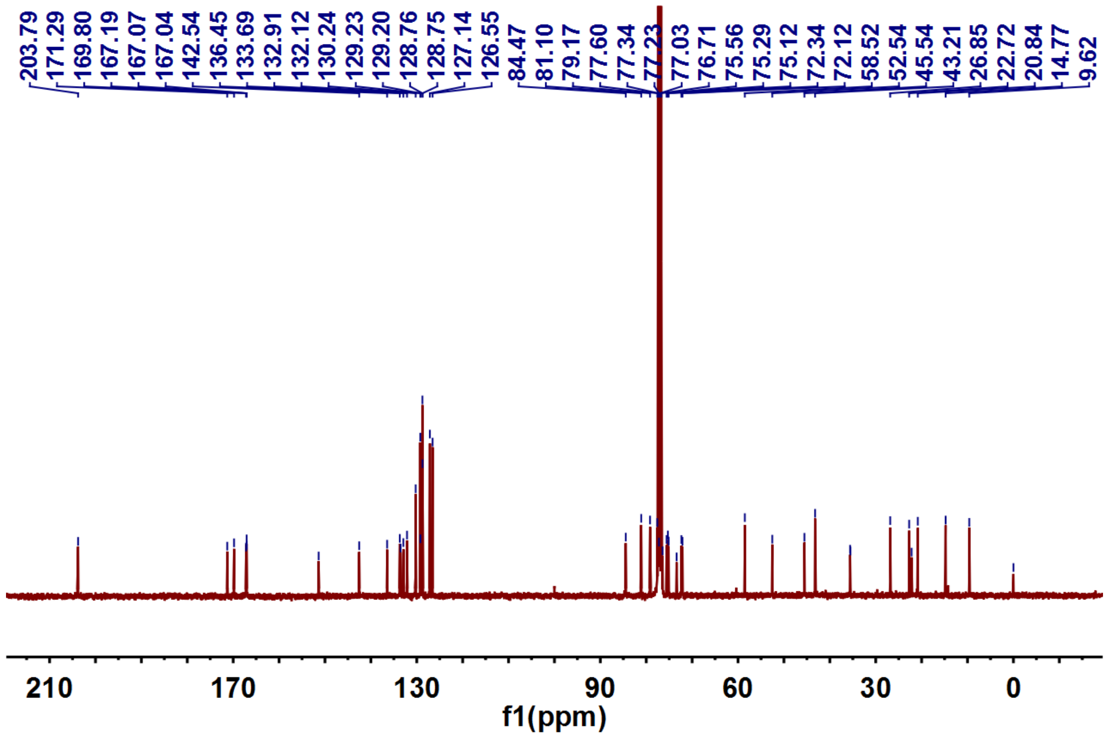

Figure S17. ^{13}C NMR spectrum of PTX-A (125 MHz, $CDCl_3$).

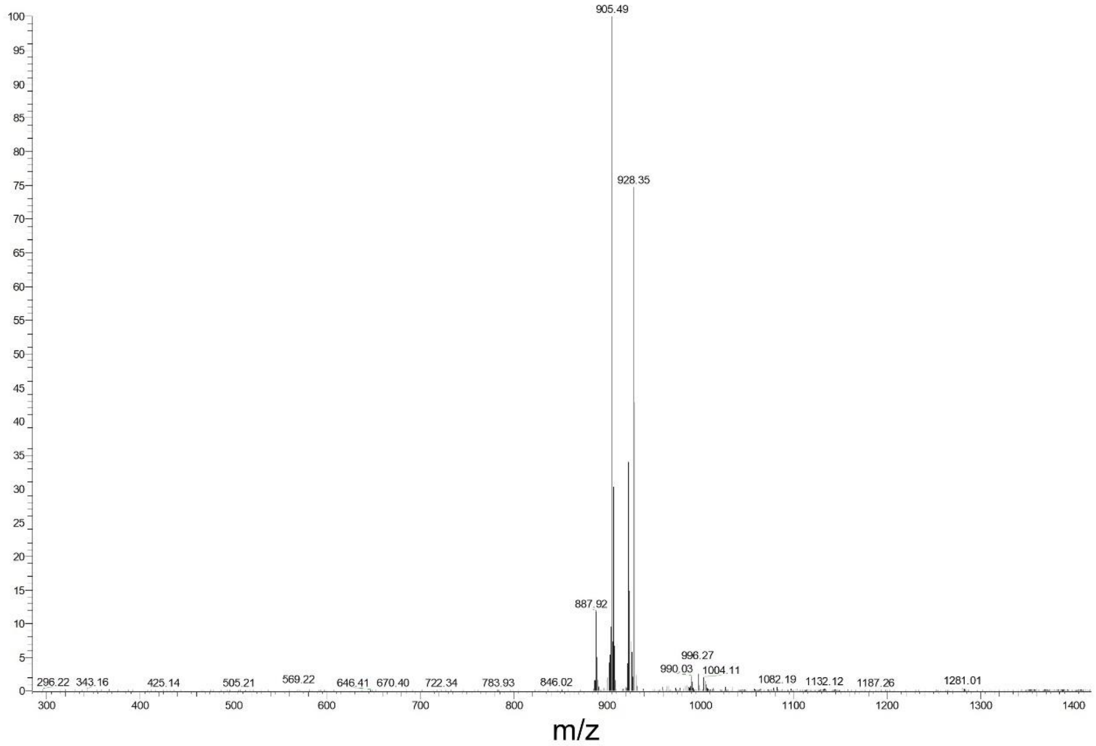

Figure S18. ESI-MS spectrum of PTX-A.

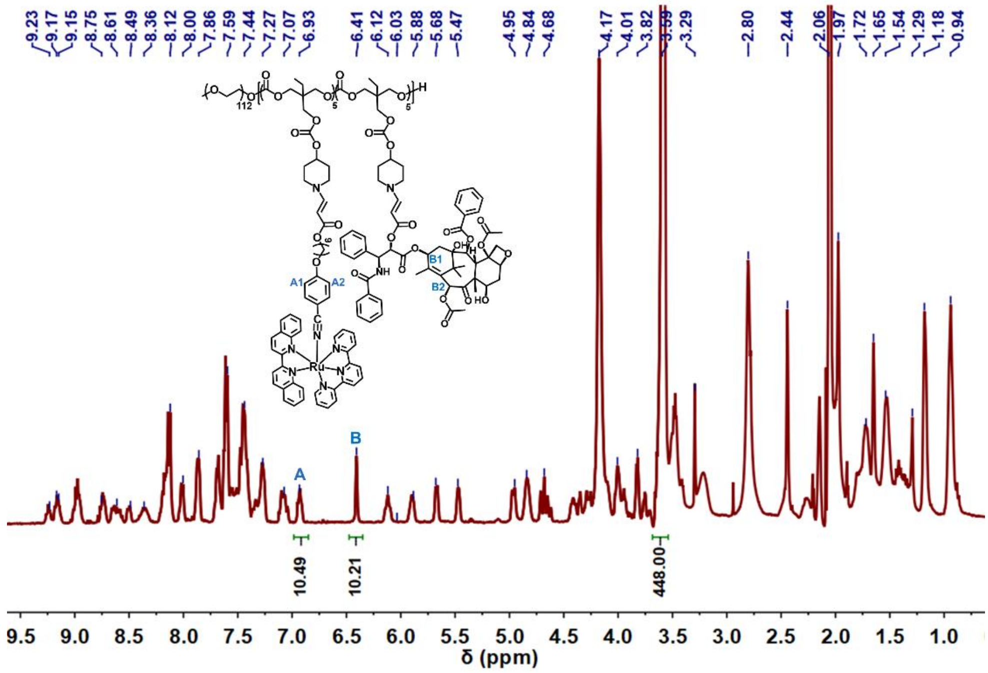

Figure S19. ^{1}H NMR spectrum of Poly(Ru/PTX) (500 MHz, $(CD_3)_2CO$). The MPEG contained 112 repeating units of $-(CH_2-CH_2-O)-$, which corresponds to 448 protons on each Poly(Ru/PTX) chain. Thus, the number of Ru complex unit was 5, and the number of PTX unit was 5. The molecular weight of Poly(Ru/PTX) = 5000 + 287*10

+ 1111*5 + 905*5 = 17950 g mol^{-1}.

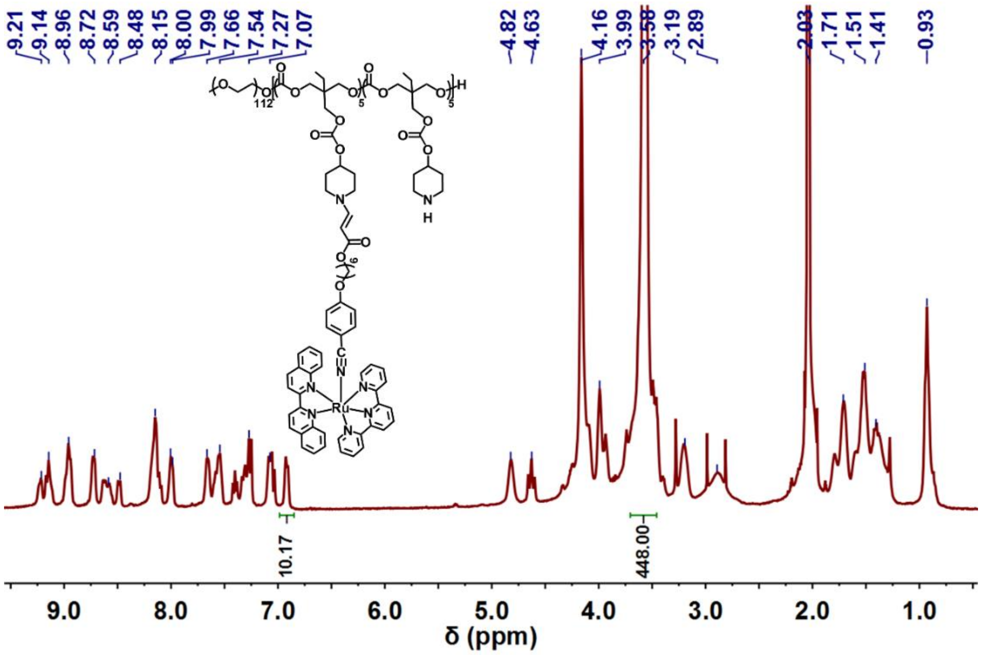

Figure S20. ^{1}H NMR spectrum of Poly(Ru) (500 MHz, $(CD_3)_2CO$).

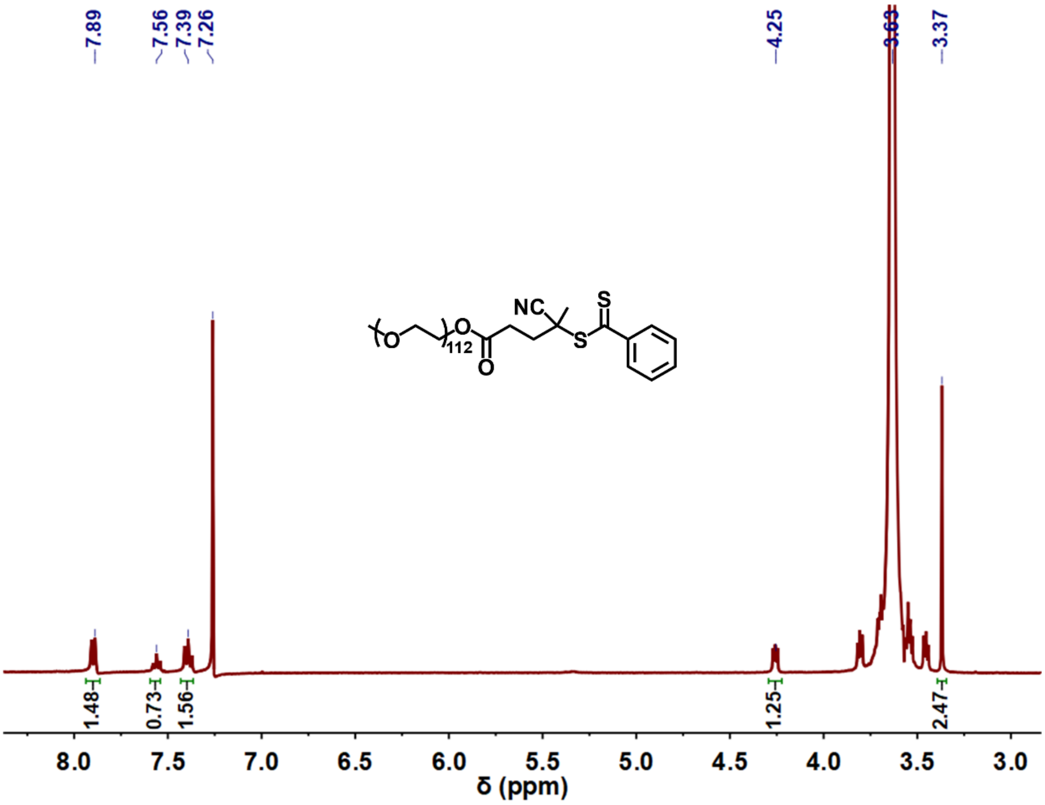

Figure S21. ^{1}H NMR spectrum of MPEGR (500 MHz, $CDCl_3$).

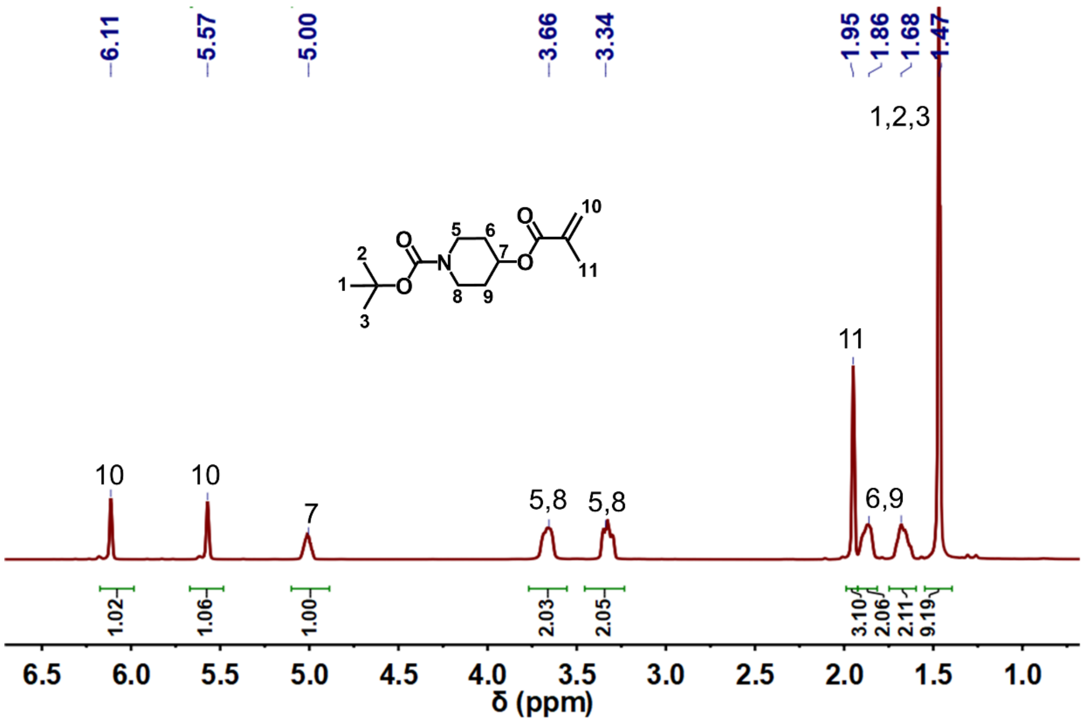

Figure S22. ^{1}H NMR spectrum of APH-Boc (500 MHz, $CDCl_3$).

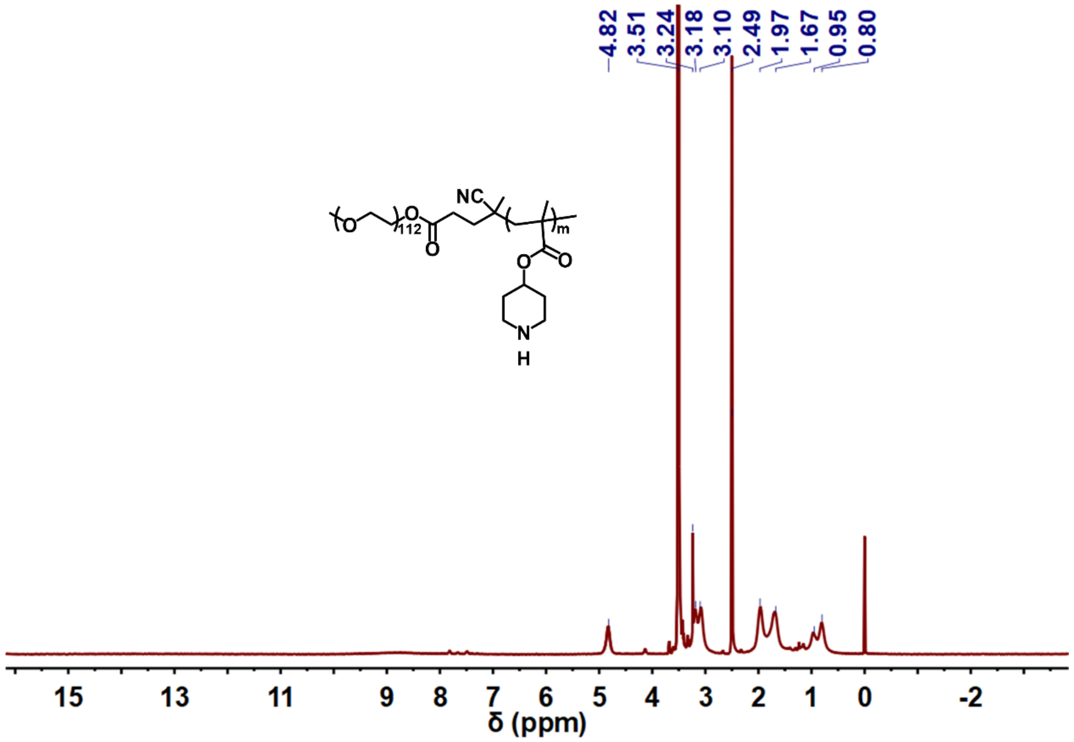

Figure S23. ^{1}H NMR spectrum of MPEG-*b*-PAPH (500 MHz, $(CD_3)_2SO$).

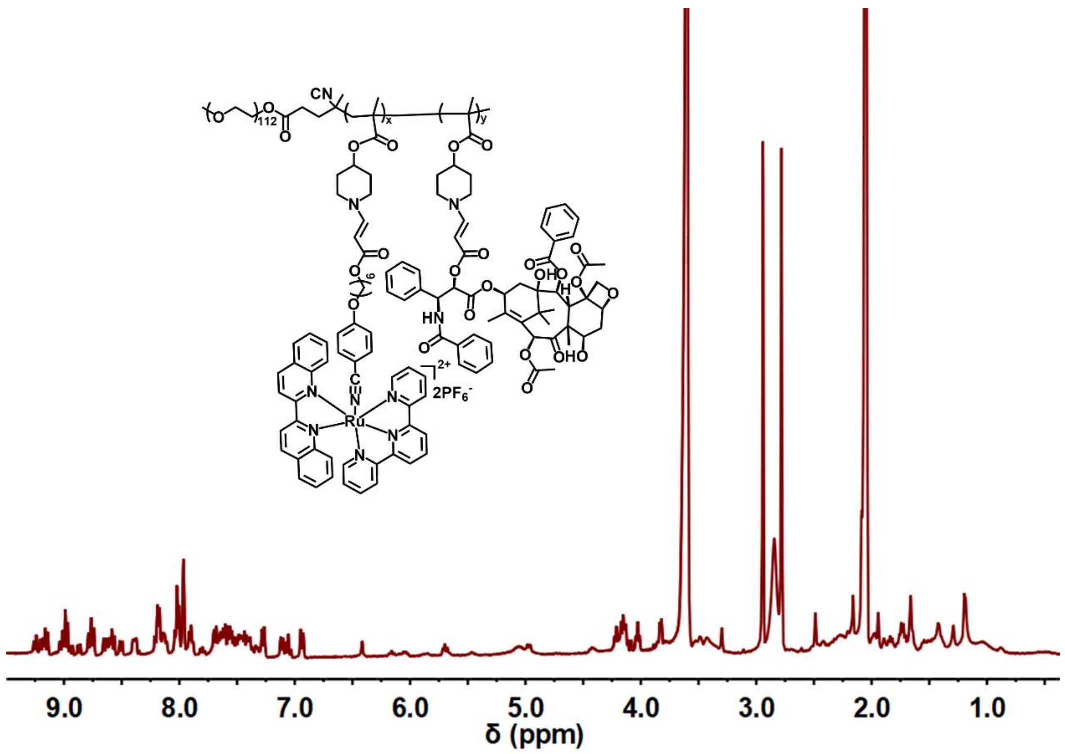

Figure S24. ^{1}H NMR spectrum of MPEG-*b*-PAPH-(Ru/PTX) (500 MHz, $(CD_3)_2CO$).

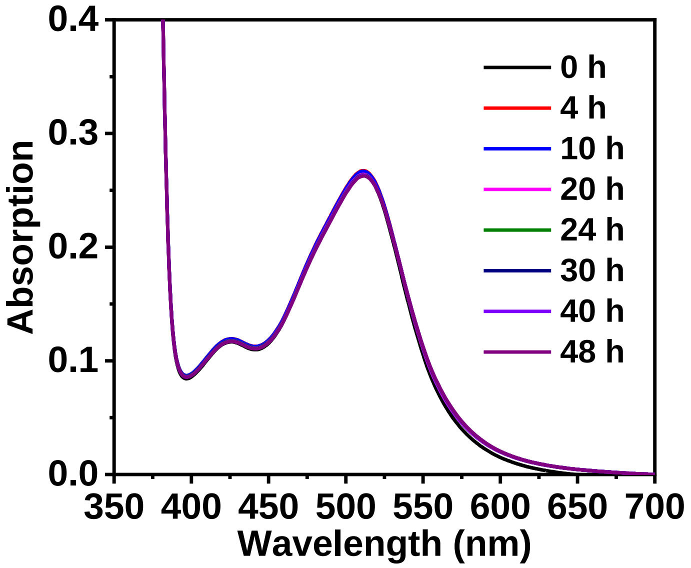

Figure S25. Absorption spectra of Poly(Ru/PTX) nanoparticles solution in dark for 48 h.

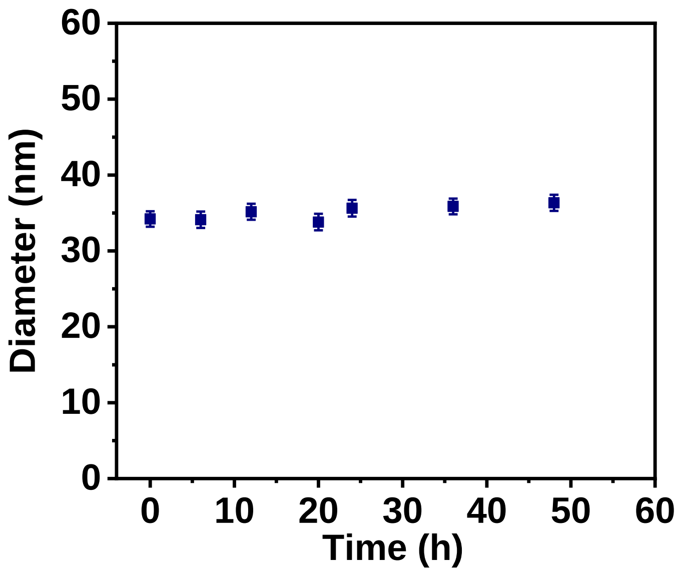

Figure S26. Diameters of Poly(Ru/PTX) nanoparticles in dark for 48 h.

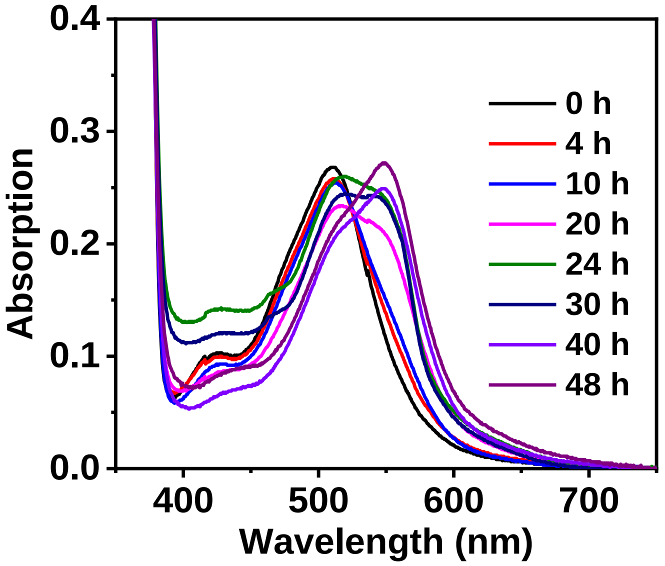

Figure S27. Absorption spectra of Ru-A solution in dark for 48 h. The metal-to-ligand charge transfer (MLCT) band at 511 nm gradually deceased and a new peak at ~549 nm appeared during incubation. Nearly half of CHA are replaced by H_2O after 24 h, suggesting that Ru-A is unstable in aqueous solution.

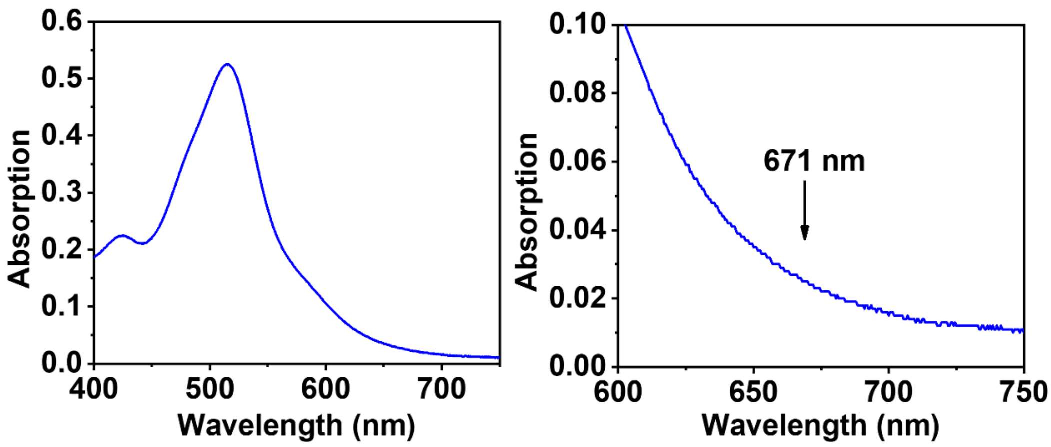

Figure S28. Absorption spectra of Poly(Ru/PTX) solution (left) and enlarged absorption spectrum (right) in 600-750 nm.

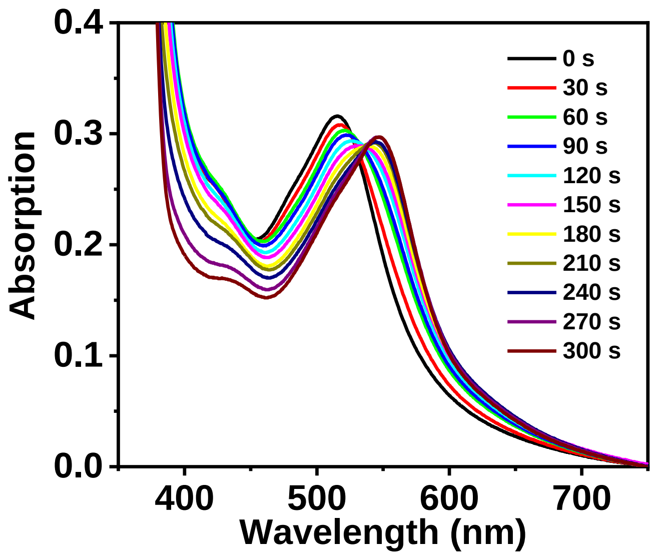

Figure S29. UV-Vis absorption spectra of Ru-A solution under red light irradiation (658 nm, 50 mW cm^{-2}) for different time periods.

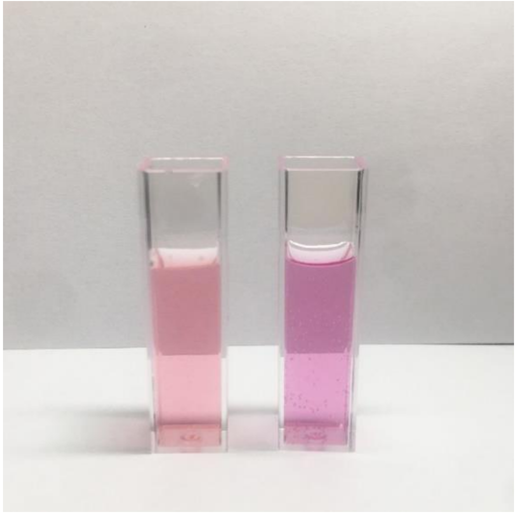

Figure S30. Photographs of Poly(Ru/PTX) nanoparticles solution before (left) and after (right) laser irradiation. The color of Poly(Ru/PTX) nanoparticles solution changed from red to purple-red due to the release of Ru-H_2O.

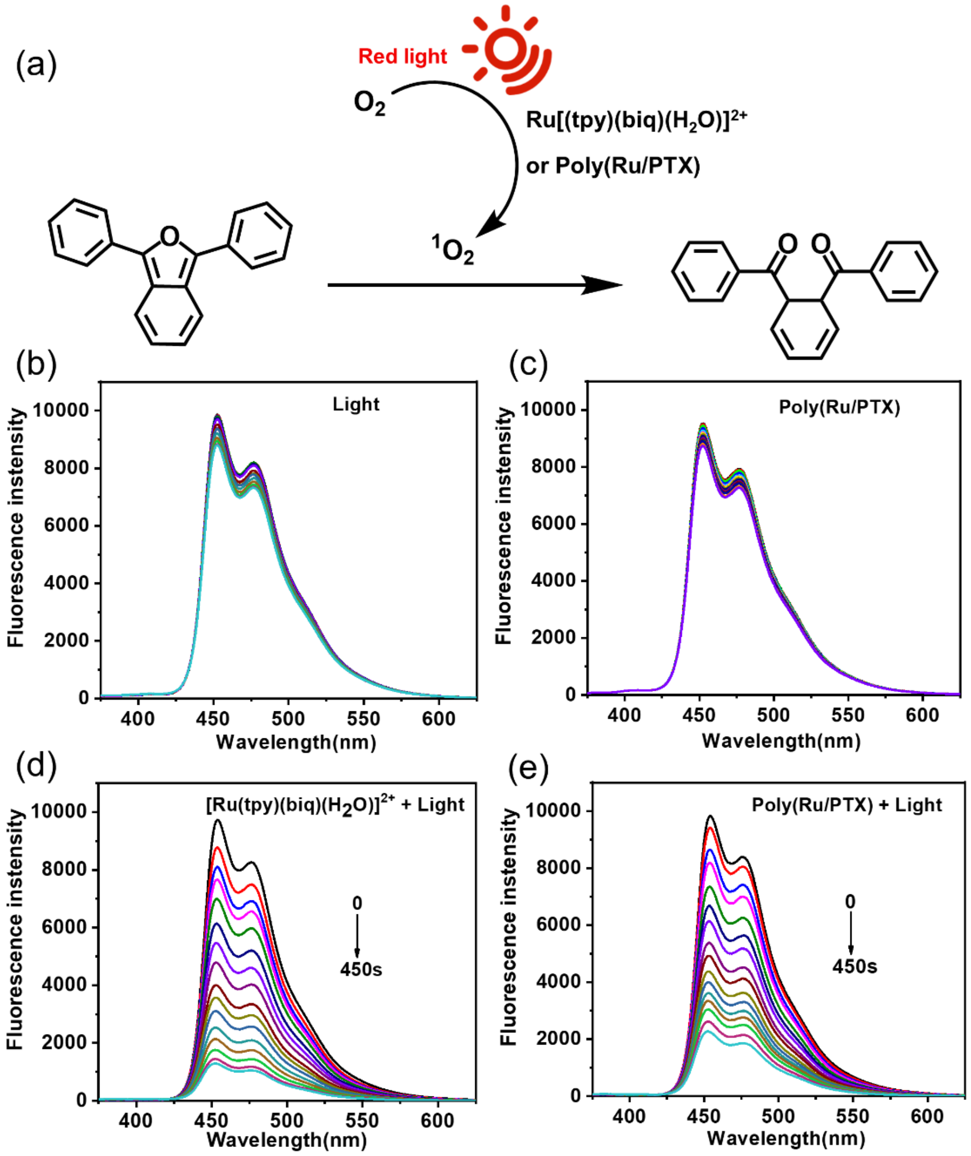

Figure S31. a) Mechanism of using 1,3-diphenylisobenzofuran (DPBF) to detect singlet oxygen ($^1O_2$) generated by Poly(Ru/PTX) under red light irradiation; b) Fluorescence spectra of DPBF (100 mM) under 671 nm light irradiation (200 mW cm^{-2}); c) Fluorescence spectra of DPBF (100 mM) in the presence of Poly(Ru/PTX) (200 μg/mL) under dark; d) Fluorescence spectra of DPBF (100 mM) in the presence of [Ru(tpy)(biq)(H_2O)]$^{2+}$ (50 μg/mL) under 671 nm light irradiation (200 mW cm^{-2}); and e) Fluorescence spectra of DPBF (100 mM) in the presence of Poly(Ru/PTX) (200 μg/mL) under 671 nm light irradiation (200 mW cm^{-2}). The estimation was according to previous literatures.[2]

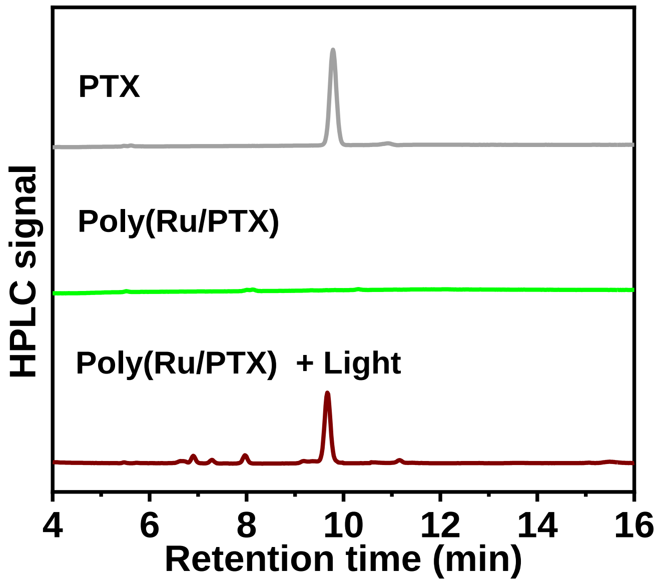

Figure S32. HPLC chromatograms of Poly(Ru/PTX) nanoparticles with or without 671 nm light irradiation (200 mW cm^{-2}, 15 min). Free PTX was set as the reference.

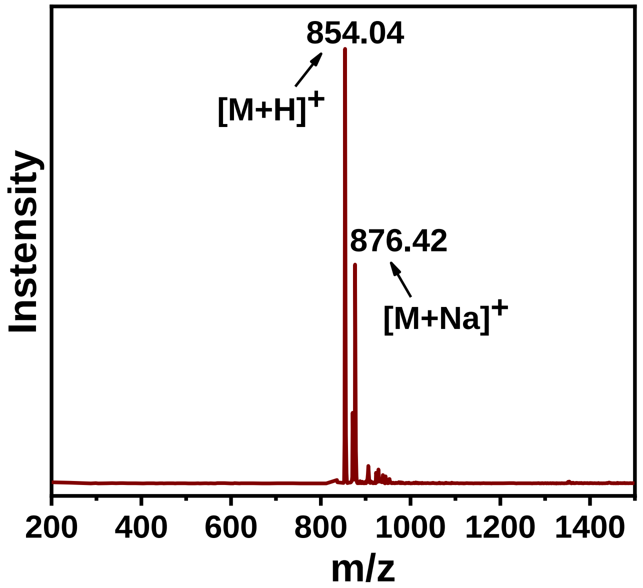

Figure S33. ESI-MS analysis of released PTX.

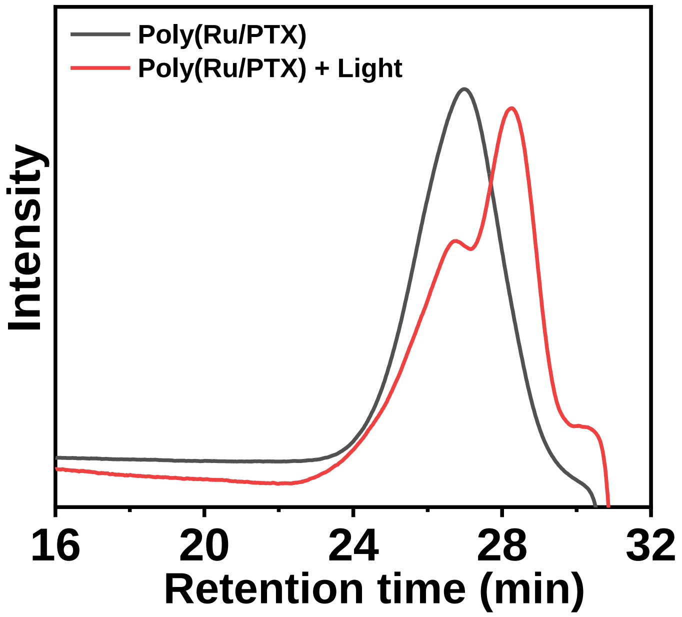

Figure S34. GPC traces of Poly(Ru/PTX) with and without light irradiation (671 nm, 200 mW cm^{-2}, 15 min).

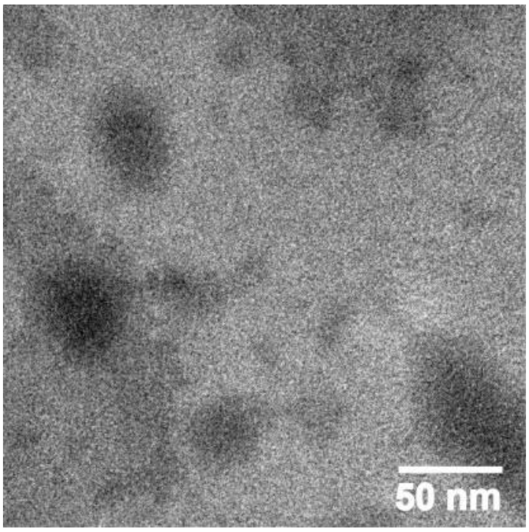

Figure S35. TEM of Poly(Ru/PTX) nanoparticles after red light irradiation.

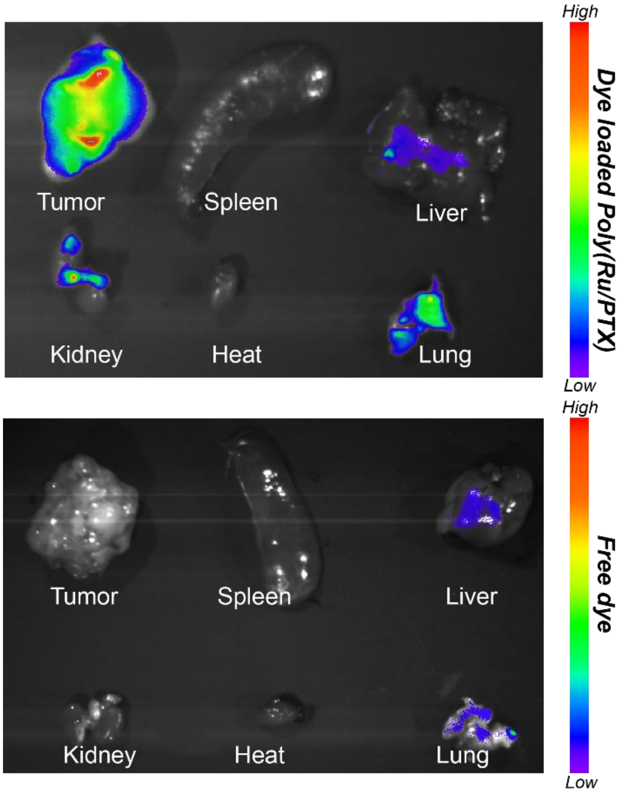

Figure S36. Ex vivo fluorescence image of major organs and tumors excised from mice injected with Free dye and dye loaded Poly(Ru/PTX) nanoparticles.

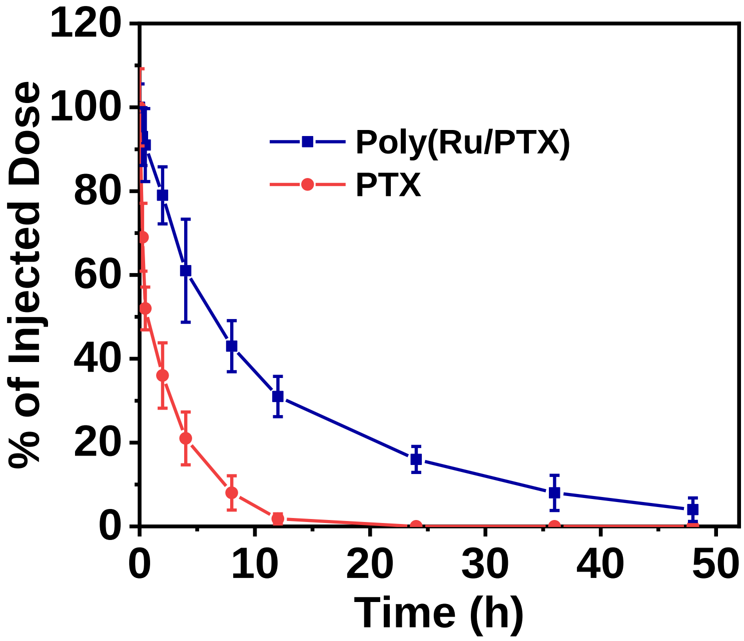

Figure S37. Plasma concentration versus time after tail vein injection of PTX and Poly(Ru/PTX) nanoparticle solutions.

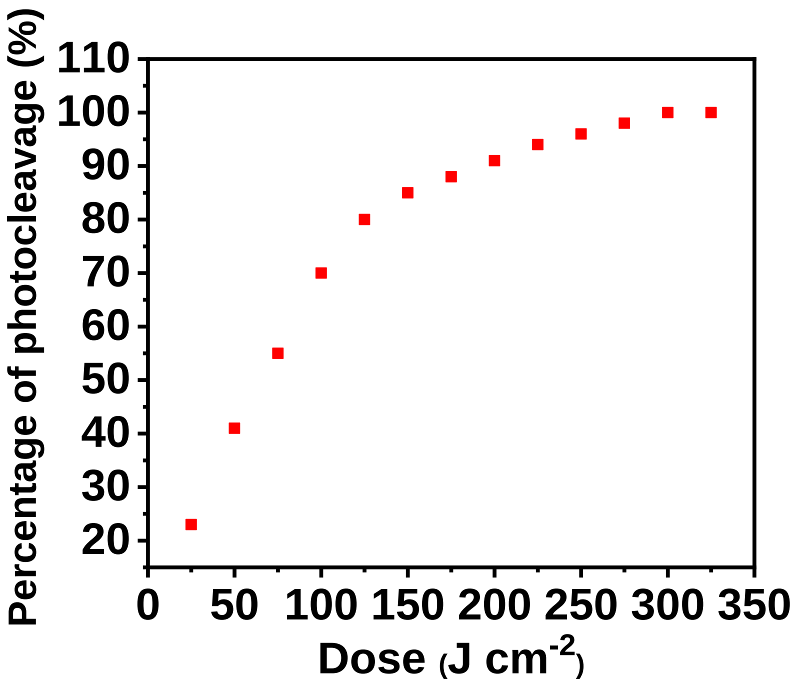

Figure S38. Irradiation dose-dependent photocleavage of Poly(Ru/PTX) estimated from the change in absorbance at λ_{max}. The estimation was according to literature.[3]

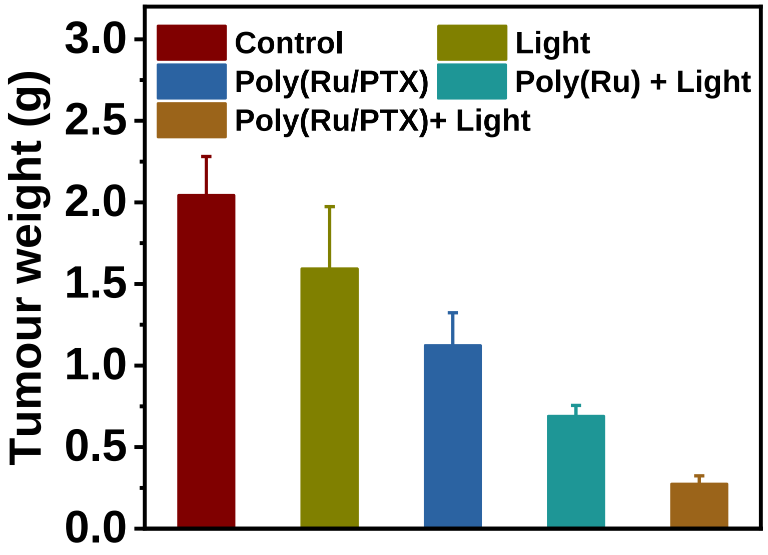

Figure S39. Average tumor weights at day 14 after different treatments.

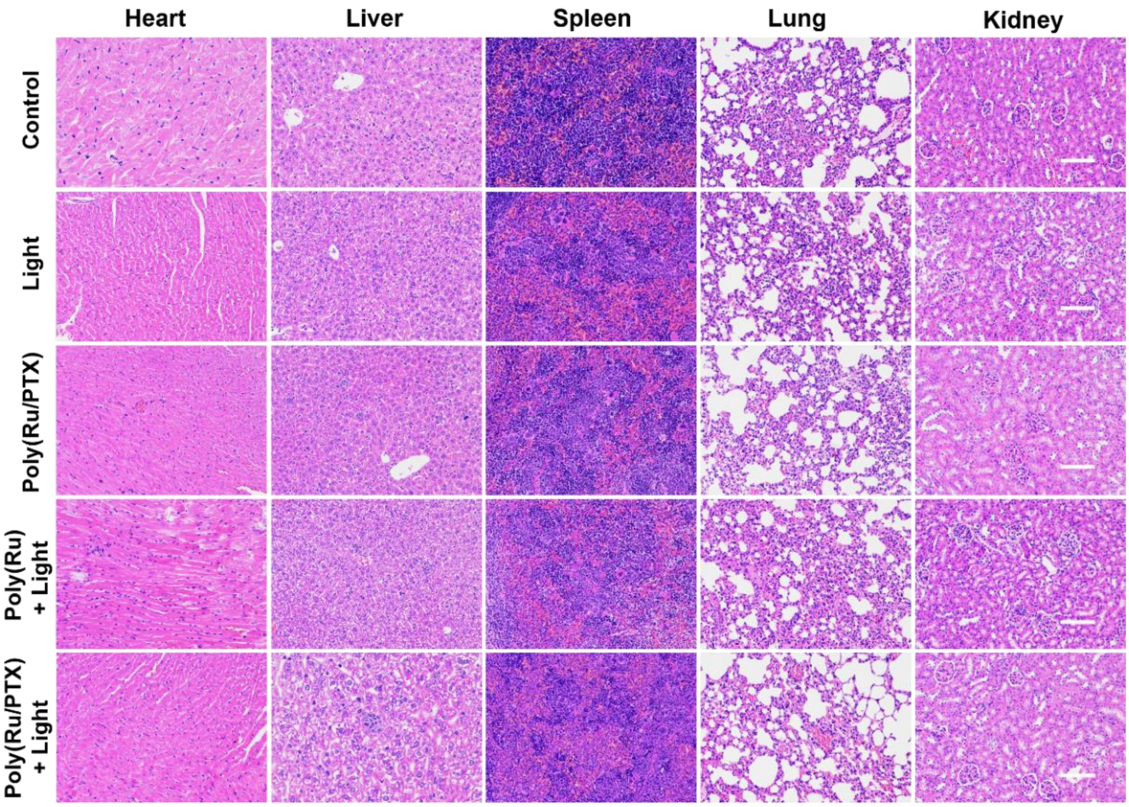

Figure S40. H&E staining of the major organs (heart, liver, spleen, lung, and kidney) tissues collected from mice in the different groups at the end of treatment. Scale bar: 100 μm.

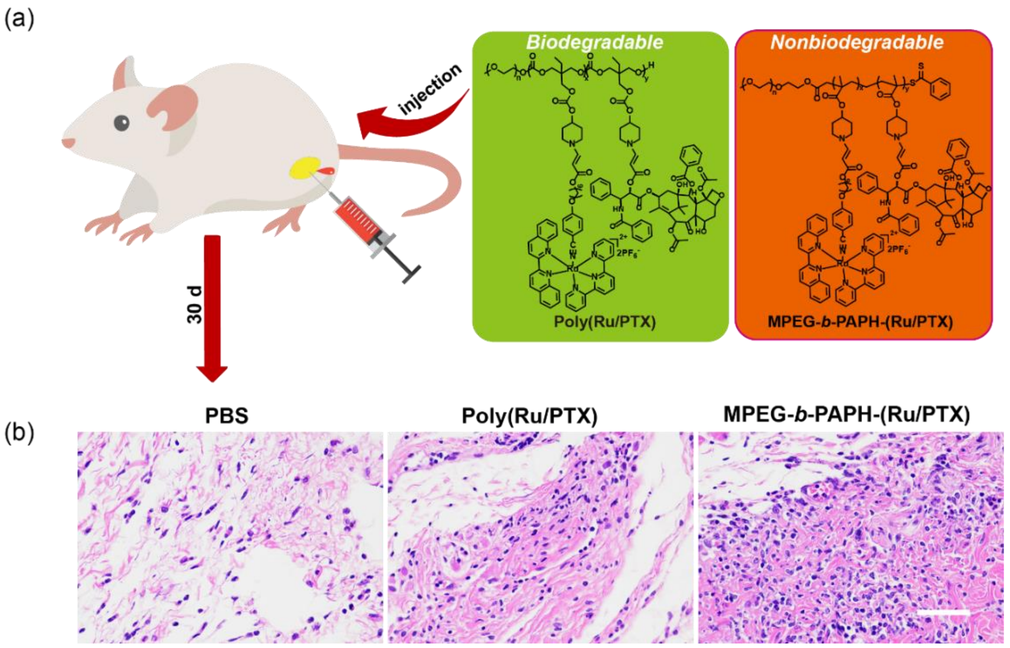

Figure S41. a) Schematic illustration of BALB/c nude mice were subcutaneously injected with PBS, Poly(Ru/PTX) nanoparticles, or MPEG-*b*-PAPH-(Ru/PTX) nanoparticles (15 mg/kg) on the right flank; b) H&E staining of formalin-fixed, paraffin-embedded skin tissues (injection sites) on day 30. Scale bar: 50 μm.

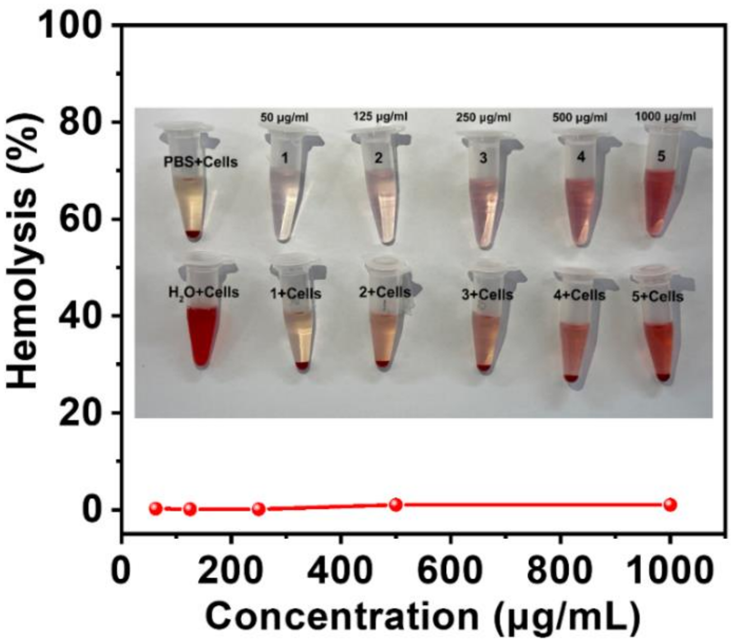

Figure S42. Blood hemolysis treated with Poly(Ru/PTX) nanoparticles at various concentrations from 62.5 μg/mL to 1000 μg/mL. Water was set as a positive control and PBS was set as a negative control.

References

[1] O. I. Kazakov, P. P. Datta, M. Isajani, E. T. Kiesewetter, M. K. Kiesewetter, *Macromolecules* **2014**, 47, 7463.

[2] a) B. A. Albani, B. Pena, N. A. Leed, N. A. de Paula, C. Pavani, M. S. Baptista, K. R. Dunbar, C. Turro, *J. Am. Chem. Soc.* **2014**, 136, 17095; b) Y. Cheng, T. L. Doane, C. H. Chuang, A. Ziady, C. Burda, *Small* **2014**, 10, 1799; c) H. He, P. C. Lo, S. L. Yeung, W. P. Fong, D. K. Ng, *J. Med. Chem.* **2011**, 54, 3097.

[3] L. Zayat, M.G. Noval, J. Campi, C. I. Calero, D. J. Calvo, R. Etchenique. *ChemBioChem* **2007**, 8, 2035.
